# Strigolactone signaling regulates specialized metabolism in tobacco stems and interactions with stem-feeding herbivores

**DOI:** 10.1371/journal.pbio.3000830

**Published:** 2020-08-18

**Authors:** Suhua Li, Youngsung Joo, Dechang Cao, Ran Li, Gisuk Lee, Rayko Halitschke, Gundega Baldwin, Ian T. Baldwin, Ming Wang

**Affiliations:** 1 Department of Molecular Ecology, Max Planck Institute for Chemical Ecology, Jena, Germany; 2 Department of Biological Sciences, Korea Advanced Institute of Science and Technology, Yuseong-gu, Daejeon, South Korea; 3 Guangdong Provincial Key Laboratory for Plant Epigenetics, College of Life Sciences and Oceanography, Shenzhen University, Shenzhen, China; 4 State Key Laboratory of Rice Biology, Institute of Insect Sciences, Zhejiang University, Hangzhou, China; 5 Department of Plant Pathology, Nanjing Agricultural University, Nanjing, China; UCSD, UNITED STATES

## Abstract

Plants are attacked by herbivores, which often specialize on different tissues, and in response, have evolved sophisticated resistance strategies that involve different types of chemical defenses frequently targeted to different tissues. Most known phytohormones have been implicated in regulating these defenses, with jasmonates (JAs) playing a pivotal role in complex regulatory networks of signaling interactions, often generically referred to as “cross talk.” The newly identified class of phytohormones, strigolactones (SLs), known to regulate the shoot architecture, remain unstudied with regard to plant–herbivore interactions. We explored the role of SL signaling in resistance to a specialist weevil (*Trichobaris mucorea*) herbivore of the native tobacco, *Nicotiana attenuata*, that attacks the root–shoot junction (RSJ), the part of the plant most strongly influenced by alterations in SL signaling (increased branching). As SL signaling shares molecular components, such as the core F-box protein MORE AXILLARY GROWTH 2 (MAX2), with another new class of phytohormones, the karrikins (KARs), which promote seed germination and seedling growth, we generated transformed lines, individually silenced in the expression of *NaMAX2*, *DWARF 14* (*NaD14*: the receptor for SL) and *CAROTENOID CLEAVAGE DIOXYGENASE 7* (*NaCCD7*: a key enzyme in SL biosynthesis), and *KARRIKIN INSENSITIVE 2* (*NaKAI2*: the KAR receptor). The mature stems of all transgenic lines impaired in the SL, but not the KAR signaling pathway, overaccumulated anthocyanins, as did the stems of plants attacked by the larvae of weevil, which burrow into the RSJs to feed on the pith of *N*. *attenuata* stems. *T*. *mucorea* larvae grew larger in the plants silenced in the SL pathway, but again, not in the *KAI2*-silenced plants. These phenotypes were associated with elevated JA and auxin (indole-3-acetic acid [IAA]) levels and significant changes in the accumulation of defensive compounds, including phenolamides and nicotine. The overaccumulation of phenolamides and anthocyanins in the SL pathway–silenced plants likely resulted from antagonism between the SL and JA pathway in *N*. *attenuata*. We show that the repressors of SL signaling, suppressor of max2-like (NaSMXL6/7), and JA signaling, jasmonate zim-domain (NaJAZs), physically interact, promoting NaJAZb degradation and releasing JASMONATE INSENSITIVE 1 (JIN1/MYC2) (NaMYC2), a critical transcription factor promoting JA responses. However, the increased performance of *T*. *mucorea* larvae resulted from lower pith nicotine levels, which were inhibited by increased IAA levels in SL pathway–silenced plants. This inference was confirmed by decapitation and auxin transport inhibitor treatments that decreased pith IAA and increased nicotine levels. In summary, SL signaling tunes specific sectors of specialized metabolism in stems, such as phenylpropanoid and nicotine biosynthesis, by tailoring the cross talk among phytohormones, including JA and IAA, to mediate herbivore resistance of stems. The metabolic consequences of the interplay of SL, JA, and IAA signaling revealed here could provide a mechanism for the commonly observed pattern of herbivore tolerance/resistance trade-offs.

## Abstract 2

Strigolactone signaling tunes specific sectors of specialized metabolism in stems of Nicotiana attenuata, such as biosynthesis of phenopropanoids and nicotine, by tailoring cross-talk among hormones, including jasmonates and auxin, to mediate changes in plant stem pigmentation and herbivore resistance to the weevil Tricobaris mucorea.

## Introduction

Plants are exposed to diverse biotic stresses, including attack from various herbivores that often specialize on different tissues. In response, plants have evolved highly sophisticated resistance strategies that involve producing different types of chemical defenses frequently targeted to different tissues. Phytohormones, including jasmonates (JAs) [1,2], ethylene [3], salicylic acid (SA) [4], auxin [5], abscisic acid (ABA), cytokinin (CK), brassinosteroids (BRs), and gibberellins (GAs) [6], have all been implicated in regulating these defenses. Among them, JAs play a pivotal role in many defensive responses [6]. The biosynthesis of JAs involves a series of enzymes, including lipoxygenases (LOXs), allene oxide cyclase (AOC), and OPDA reductase (OPR) 3 [7]. In the presence of jasmonic acid-isoleucine (JA-Ile), jasmonate zim-domain (JAZ) proteins are recruited by CORONATINE INSENSITIVE 1 (COI1) in a S-phase kinase-associated protein 1 (Skp1)–Cullin–F-box (SCF) E3 ubiquitin ligase complex and further degraded by the 26S proteasome pathway [7]. *Nicotiana attenuata*, a wild tobacco species native to the Great Basin Desert in the United States and an ideal ecological model plant for the study of plant–herbivore interactions, produces many JA-mediated secondary metabolites that function as direct and indirect defenses, such as nicotine [8,9], trypsin proteinase inhibitors (TPIs) [10], phenolic chlorogenic acid (CGA) [11], phenolamides [12,13], 17-hydroxygeranyllinalool diterpene glycosides (HGL-DTGs) [14], and volatile organic compounds [15].

Although most classes of phytohormones have been intensively studied with regard to their roles in herbivore defense, the newly identified class of plant hormones, strigolactones (SLs), remains unstudied in this regard. SLs are known to regulate plant growth and development, such as shoot and root architecture [16]. SLs are mainly synthesized in roots and transported to shoots or exuded into the rhizosphere, where they function as hormones or external signals [16–18]. More than 20 SLs have been characterized in a range of plant species, including *Arabidopsis thaliana*, *Oryza sativa*, *Solanum lycopersicum*, *Petunia hybrida*, and *N*. *tabacum* [19]. The biosynthetic pathway of SLs has been elucidated as a result of the identification of SL-deficient mutants, which all display highly branched phenotypes [16]. SLs are derived from carotenoids. Enzymes including DWARF 27 (D27), carotenoid cleavage dioxygenases 7 and 8 (CCD7 and CCD8), MORE AXILLARY GROWTH 1 (MAX1), and lateral branching oxidoreductase (LBO) function consecutively in the biosynthetic pathway to produce SLs [16,20].

SL perception requires binding to an α/β-hydrolase superfamily protein, DWARF 14 (D14), which contains a canonical catalytic triad and functions in both SL hydrolysis and SL signal transduction [16,21]. In the presence of SLs, or the synthetic SL analogue *rac*-GR24, AtD14/D14 interacts with an F-box protein, AtMAX2/D3. Similar to *d14* mutants, *max2*/*d3* mutant plants exhibit dwarfed and highly branched or high-tillering shoot architectures, with distinct root–shoot junctions (RSJs), which cannot be rescued by exogenous applications of SL analogues [22]. Moreover, these SL-insensitive mutants are exuberant producers of SLs, likely because of the negative feedback of SL perception on SL biosynthesis [16], as has been reported from studies of the JA and ethylene signaling pathways in *N*. *attenuata* [3,23]. MAX2 is shared by both SL and karrikin (KAR) signaling pathways [24]. KARs are a group of butenolide compounds characterized in smoke from wildfires, which are perceived by karrikin-insensitive (KAI2) and stimulate seed germination in many plant species, as well as morphological responses in *Arabidopsis* [25]. Therefore, *max2*/*d3* mutants show additional phenotypes regulated by KAR signaling that are not observed in *d14* or SL-deficient mutants, such as enhanced seed dormancy and resistance to hypocotyl inhibition by KAR treatments [24]. The SL-mediated interaction of AtD14-AtMAX2 in *Arabidopsis* or D14-D3 in rice further triggers the degradation of three repressors, SUPPRESSOR OF MAX2-LIKE (SMXL) 6, SMXL7, and SMXL8 in *Arabidopsis* or D53 in rice [26].

Signaling interactions among various phytohormones is often generically referred to as “cross talk,” and various types of cross talk of SLs with other hormones, with both direct and indirect interactions, have been reported. In regulating axillary bud outgrowth, transcripts of SL biosynthetic genes are up-regulated by auxin [27]. Conversely, SLs rapidly influence the polarization of auxin efflux carrier pin-formed (PIN) 1, which impairs auxin transport and consequently impedes the outgrowth of axillary buds in *Arabidopsis* [28,29]. Similarly, during the SL-mediated regulation of root architecture, SL biosynthesis is stimulated by auxin in roots through the up-regulation of *CCD7* and *CCD8*, and SLs likely modulate auxin flux to regulate root development, as it has been shown that GR24 treatment decreases PIN1, PIN3, and PIN7 protein expression in roots [30]. In addition to the interaction with auxin signaling, SLs and CK act antagonistically by regulating the transcription of *BRANCHED 1* (*BRC1*), the central bud outgrowth mediator in axillary buds [31,32]. Furthermore, MAX2 directly interacts with the BR transcriptional effector *bri1*-EMS-SUPPRESSOR 1 (BES1), leading to accelerated BES1 degradation [33]. Cross talk between SL and ethylene signaling has also been reported. In the ethylene signaling mutants *ethylene receptor* (*etr*) and *ethylene insensitive* (*ein*), SL responses are attenuated, and the ethylene biosynthetic gene *1-AMINOCYCLOPROPANE-1-CARBOXYLIC ACID SYNTHASE 2* (*ACS2*) is up-regulated in response to GR24 treatments, indicating synergistic effects between SLs and ethylene signaling [30]. Lastly, D14 can physically interact with a GA signaling repressor, PSEUDOGENE OF S-LOCUS RELATED PROTEIN (SLR1), in an SL-dependent manner [34]. GA signaling has been reported to reduce SL production in root exudates and subsequently reduces seed germination of parasitic plants [35].

In addition to regulating plant development, SLs also play an important role in plant resistance to abiotic and biotic stresses. Ha and colleagues found that SLs act positively in regulating responses to drought and salt stress in *Arabidopsis* [36]. *Arabidopsis max2* mutants are more susceptible to the attack from two kinds of bacterial pathogens [37], and the SL-deficient tomato mutant *Slccd8* is more susceptible to the foliar fungal pathogens *Botrytis cinerea* and *Alternaria alternata* [38]. Recently, two groups reported opposing roles of SLs on plant defense in tomato and in rice against root-knot nematodes (RKNs) [39,40]. Whether SLs play a role in deterring attack from insect herbivores is largely unknown.

Here, we explore the role of SL and KAR signaling in plant defense against an endophytic herbivore, *Trichobaris mucorea*, which is a native stem-boring herbivore of *N*. *attenuata* in the Great Basin Desert of the US. *T*. *mucorea* larvae feed on the pith of stems and enter the plant just after hatching at their oviposition sites in the RSJ to complete their 4-month life cycle inside the stems of plants, which includes four larval instars, pupation, and emergence into adults [11]. This larval herbivore consumes only the pith of plants, with few externally visible signs of its attack other than a telltale accumulation of anthocyanin on the stem during stem elongation and an exit hole in the stem when the plant has senesced, which allows the adult to leave the stem, mate, and after overwintering in the duff, oviposit into another generation of plants in the following growing season [41].

We became interested in the role of SL signaling in resistance to this specialist herbivore because of its attack of the RSJ, the part of the plant most strongly morphologically influenced by alterations in SL signaling (increased branching), and its elicitation of anthocyanin pigments in stems. We generated 10 independently transformed lines each silenced in the expression of *NaMAX2* (*max2*), *NaD14* (*d14*), *NaCCD7* (*ccd7*), and *NaKAI2* (*kai2*) and selected two lines from each set of transformed plants that harbored single complete insertions of the RNA interference (RNAi) transgene construct and were efficiently silenced in the target gene in the homozygous T_2_ generation. As expected, *max2*, *d14*, and *ccd7* plants produced more primary branches, but unexpectedly, the stems of these plants were red pigmented because of their high anthocyanin levels. We found that *max2*, *d14*, and *ccd7* plants were more susceptible to attack from *T*. *mucorea* larvae, producing significantly larger larvae, and were chosen by larvae in split-stem choice bioassays. By transcriptomics and untargeted metabolomics analyses, we discovered that the RSJ of SL-RNAi plants, but not *kai2* plants, had higher levels of JA, indole-3-acetic acid (IAA), and the proven foliar defensive metabolites, phenolamides, but lower levels of nicotine. Further analyses revealed that the physical interactions of NaSMXL6/7 and NaJAZs might be responsible for the enhanced JA responses, which in turn increased the production of phenolamides and anthocyanins. However, the greater susceptibility of SL-RNAi plants to *T*. *mucorea* attack, despite their enhanced JA signaling, results from the plants’ lower nicotine levels, which in turn result from higher auxin levels. This auxin-dependent regulation of nicotine accumulation and resulting larval performance was confirmed by decapitation and auxin transport inhibition. These results provide direct evidence that SL signaling plays a positive role in regulating defense against the stem-boring herbivore *T*. *mucorea* by interacting with JA and auxin to mediate nicotine biosynthesis. The research highlights the complexity of real plant–herbivore interactions that are frequently mediated by equally complex phytohormonal cross talk, the mechanisms of which still need to be elucidated.

## Results

### *max2*, *d14*, and *ccd7* plants, but not *kai2* plants, produce more branches with stems that accumulate higher levels of anthocyanins

**Fig 1:**
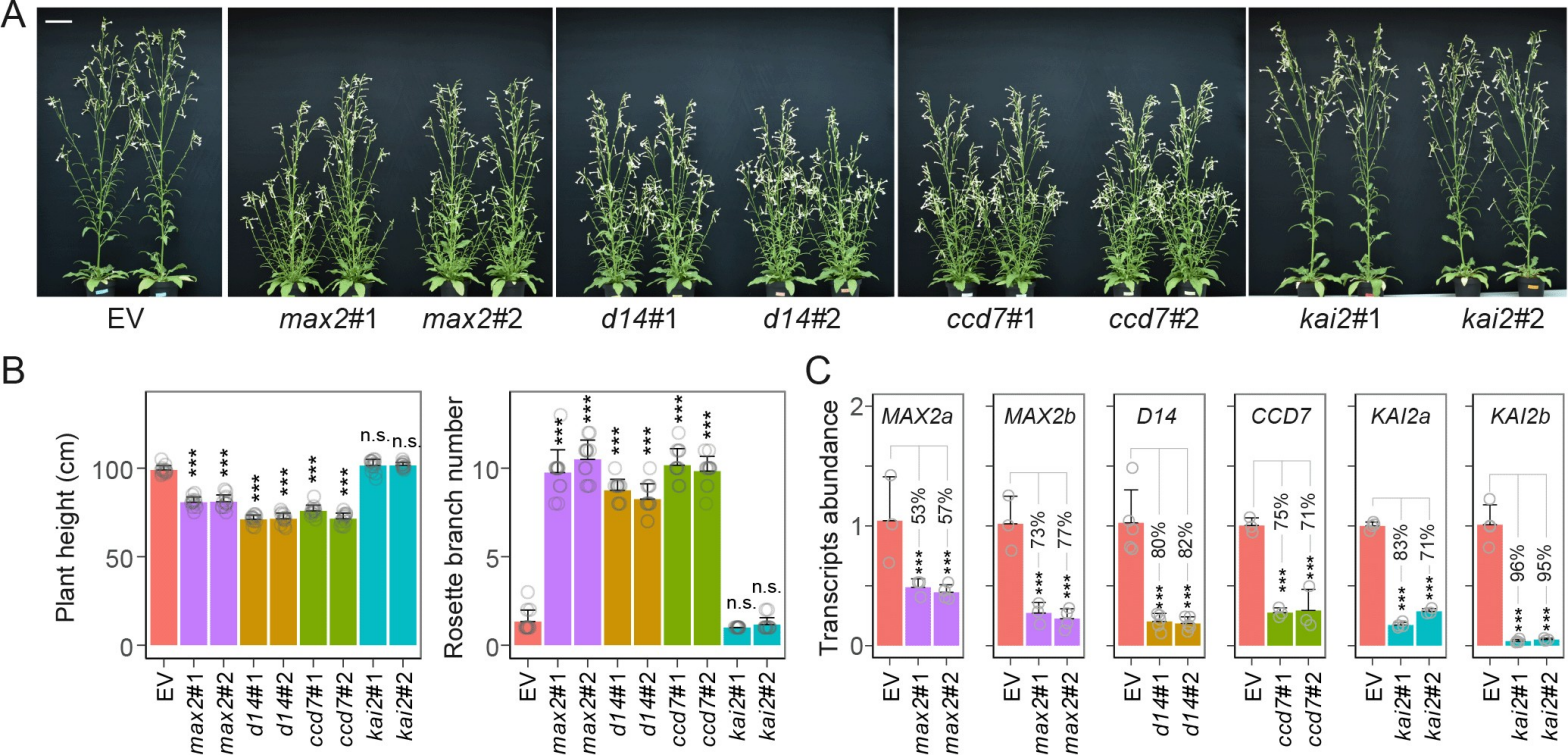
SL-RNAi plants have more branches. (A) Representative branching phenotypes of EV, two independent transgenic lines in which the target genes were individually silenced: *max2*#1 and *max2*#2, *d14*#1 and *d14*#2, *ccd7*#1 and *ccd7*#2, and *kai2*#1 and *kai2*#2 plants. Scale bar, 5 cm. (B) Plant height and primary branch numbers per plant of indicated plants (±SE, *n =* 12). Branches longer than 5 cm were counted. (C) Silencing efficiency of target genes. Relative transcript abundance of *MAX2a* and *MAX2b* in axillary buds of EV, *max2*#1, and *max2*#2 plants; *D14* in axillary buds of EV, *d14*#1, and *d14*#2 plants; *CCD7* in seedlings of EV, *ccd7*#1, and *ccd7*#2 plants; *KAI2a* and *KAI2b* in leaves of EV, *kai2*#1, and *kai2*#2 plants (±SE, *n =* 3–4) (***P < 0.001; two-tailed Student *t* test). Values for graphs (B) and (C) are listed in S1 Data. ccd, carotenoid cleavage dioxygenase; d14, dwarf 14; EV, empty vector; kai2, karrikin insensitive 2; max2, more axillary growth 2; n.s., not significant; RNAi, RNA interference; SL, strigolactone.

To explore the role of SL and KAR signaling in the plant’s resistance to stem-boring herbivores, we first characterized *MAX2*, the coreceptor of both SL and KAR signaling, in *N*. *attenuata*. The two homologues of MAX2, NaMAX2a, and NaMAX2b, were found to be localized to the nucleus (S1A and S1B Fig), and both proteins interacted with NaD14 in the presence of GR24 (S1C Fig). A virus-induced gene silencing (VIGS) experiment that independently silenced the two homologues, albeit relatively weakly (with 61% of *MAX2a* and 63% of *MAX2b* silencing efficiency, respectively) suggested functional redundancy of the two homologues, as the characteristic branching phenotype of SL-deficient lines (Fig 1A and 1B) was not observed. Given that both proteins interacted with D14, we next generated a stable transgenic line expressing an RNAi construct, inverted repeat *max2*, which silenced both *MAX2a* and *MAX2b* with a single inverted repeat tandem construct (S1D Fig). Two independent homozygous transgenic lines, *max2*#1 and *max2*#2 (Fig 1A), each harboring single, complete insertions, as verified by several NanoString probes (S1E Fig), were selected. As the silencing efficiency of *MAX2a/b* was generally low (50%–70% silencing efficiency) (Fig 1C), phenotyping assays in addition to the stem/branch number assays (Fig 1B) were conducted to characterize the *max2* lines in greater depth: in addition to increased branching, both lines of *max2* plants showed attenuated responses to GR24 and KAR treatments in assays that measured hypocotyl elongation and seed germination (S1F and S1G Fig). Three additional suites of transgenic lines in which the key components of the SL and KAR signaling pathways, including *D14*, *CCD7*, and *KAI2* genes (S2A and S2B Fig), were silenced by RNAi were generated (Fig 1A and 1C). For each silencing construct, we selected two independent transgenic lines, which harbored a single complete insertion of the inverted repeat RNAi construct, as verified by several NanoString probes (S2C–S2E Fig) [42]. A transgenic line transformed with an empty vector (EV) was used as control plants [11]. All lines of SL-insensitive (*max2* and *d14*) and SL-deficient (*ccd7*) plants displayed increased numbers of primary branches and shorter plant heights; two *kai2* lines did not differ from control plants in these parameters (Fig 1A and 1B). Consistent with a negative-feedback loop controlling SL production, transcripts of two SL biosynthetic genes, *NaCCD7* and *NaCCD8*, were up-regulated in roots of *max2* and *d14* plants (S3A and S3B Fig). When plants were grown under low phosphate supply rates, the levels of (±)2′-*epi*-orobanchol, the active form of SL in *N*. *attenuata*, were approximately 20- to 50-fold higher in the root exudates of *d14* and *max2* plants (S3C–S3F Fig); in contrast, this compound was nearly undetectable in the root exudates of *ccd7* plants (S3F Fig).

**Fig 2:**
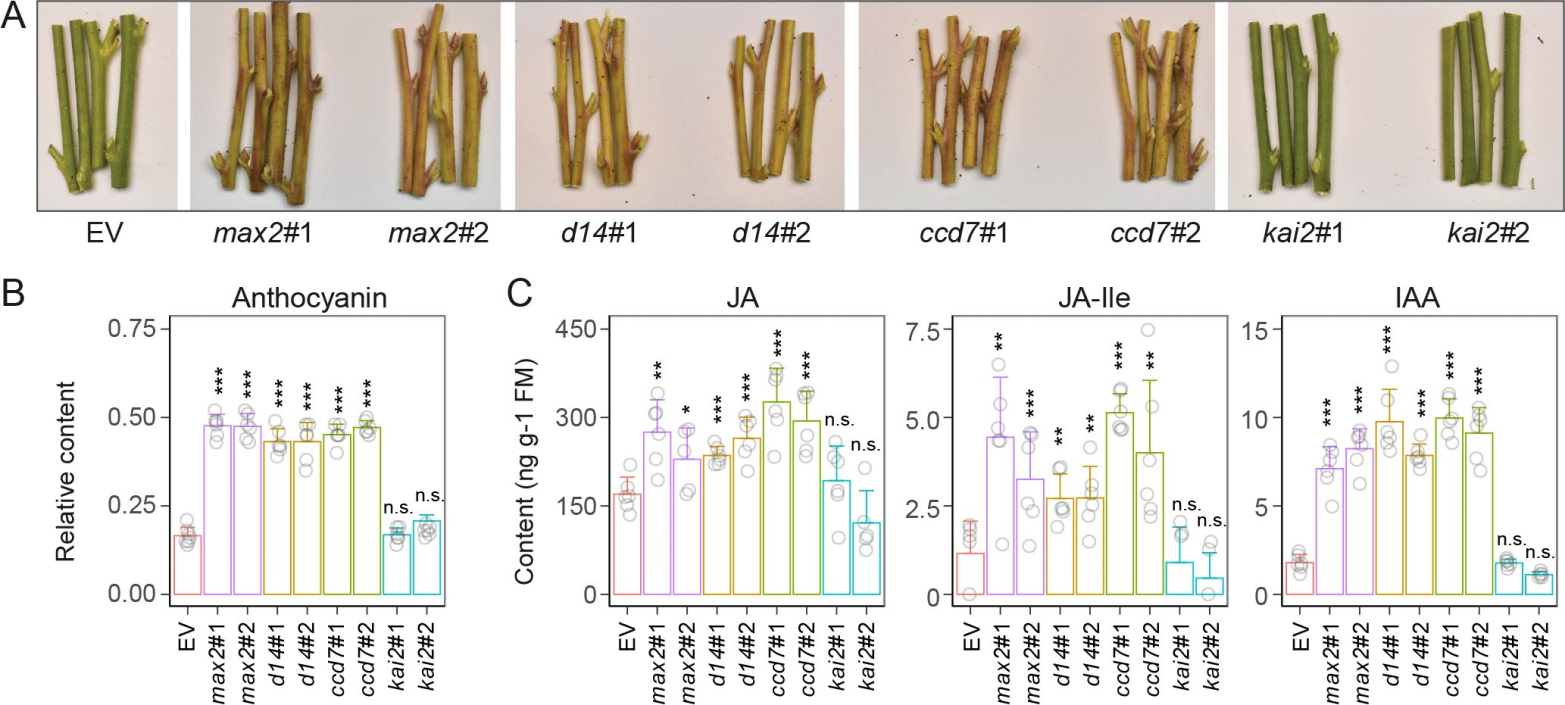
Anthocyanin accumulations in stems of *max2*, *d14*, *ccd7*, and *kai2* plants. (A) Representative stem phenotypes of 75-day-old EV, *max2*, *d14*, *ccd7*, and *kai2* plants. (B) Relative anthocyanin levels in epidermis of stems of indicated plants (±SE, *n =* 8). (C) Levels of JA, JA-Ile, and IAA in stems of indicated plants (±SE, *n =* 6) (*P < 0.05; **P < 0.01; ***P < 0.001; two-tailed Student *t* test). Values for graphs (B) and (C) are listed in S1 Data. ccd, carotenoid cleavage dioxygenase; d14, dwarf 14; EV, empty vector; IAA, indole-3-acetic acid; JA, jasmonate; JA-Ile, jasmonic acid-isoleucine; kai2, karrikin insensitive 2; max2, more axillary growth 2; n.s. not significant.

Interestingly, the stems of *max2*, *d14*, and *ccd7* plants turned red at the later developmental stages of flowering (Fig 2A), a phenotype not previously reported in other SL-related studies. Accordingly, the anthocyanin levels were significantly higher (2-fold) in these three SL-RNAi plants (Fig 2B). Given the role of JAs and auxin in the regulation of anthocyanin accumulations in *N*. *attenuata* [5], the constitutive levels of JAs (JA and JA-Ile) and IAA were quantified. Both JA and IAA levels were significantly increased in the stems of *max2*, *d14*, and *ccd7* plants, but the fold change of IAA was overall greater than that of JAs (Fig 2C). However, neither stem color and anthocyanin content nor JA and IAA levels in *kai2* plants differed significantly from those of EV control plants (Fig 2).

### *max2*, *d14*, and *ccd7* plants, but not *kai2* plants, are more susceptible to the larvae of the stem-boring weevil, *T*. *mucorea*

**Fig 3:**
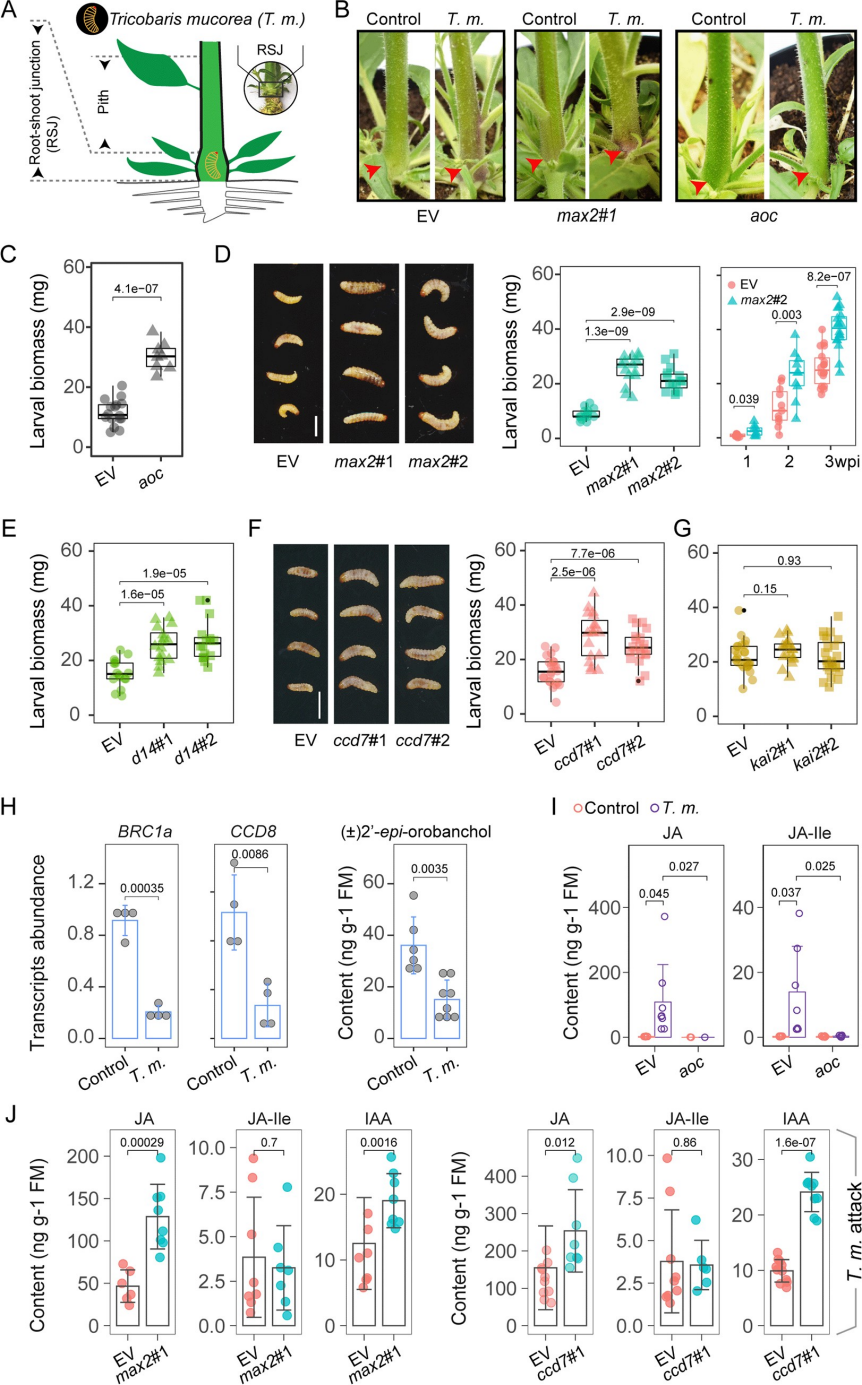
*max2*, *d14*, and *ccd7* plants are more susceptible to *T*. *m*. attack. (A) Schematic diagram of a *T*. *m*.*–*attacked plant. Stem pith and RSJ part used in this study are marked. (B) Representative pictures of EV, *max2*, and *aoc* plants after 3 weeks of *T*. *m*. attack; red pigmentation occurred along stems after attack or in *max2* plants regardless of attack as indicated by red arrows. (C) Biomass of *T*. *m*. larvae after attacking EV and *aoc* plants for 2 weeks (*n =* 8–15). (D) Representative pictures of *T*. *m*. larvae after attacking EV, *max2*#1, and *max2*#2 plants for 2 weeks and biomass of *T*. *m*. larvae after attacking EV, *max2*#1, and *max2*#2 plants for 2 weeks (*n =* 15). Kinetic analysis of *T*. *m*. larvae biomass accumulations after 1, 2, and 3 wpi (*n =* 10–18). Scale bar, 2 mm. (E) Biomass of *T*. *m*. larvae after attacking EV, *d14*#1, and *d14*#2 plants for 2 weeks (*n =* 16). (F) Representative pictures and biomass of *T*. *m*. larvae after attacking EV, *ccd7*#1, and *ccd7*#2 plants for 2 weeks (*n =* 20). (G) Biomass of *T*. *m*. larvae after attacking EV, *kai2*#1, and *kai2*#2 plants for 3 weeks (*n =* 20). (H) Relative transcript abundance of *BRC1a* and *CCD8* in stem pith of EV plants without (Control) or with *T*. *m*. attack (*T*. *m*.) for 2 weeks (±SE, *n =* 4). Levels of (±)-2′-*epi*-orobanchol in roots with indicated treatments (±SE, *n =* 8). (I) Levels of JA and JA-Ile in piths of EV and *max2* plants without or with *T*. *m*. attack for 3 weeks (±SE, *n =* 6–10). (J) Levels of JA, JA-Ile, and IAA in piths of EV, *max2*, and *ccd7* plants with *T*. *m*. attack for 2 weeks (±SE, *n =* 6–10) (two-tailed Student *t* test). Values for graphs (C–J) are listed in S1 Data. aoc, allene oxide cyclase; BRC1, branched 1, ccd, carotenoid cleavage dioxygenase; d14, dwarf14; EV, empty vector; IAA, indole-3-acetic acid; JA, jasmonate; JA-Ile, jasmonic acid-isoleucine; kai2, karrikin insensitive 2; max2, more axillary growth 2; n.s. not significant; RSJ, root–shoot junction; *T*. *m*., *T*. *mucorea*; wpi, weeks postinoculation.

The greater anthocyanin contents of the stems and the higher JA levels of the SL-RNAi plants suggested alterations in plant defense in these lines [1,43]. To explore this inference, we investigated the role of SL signaling in herbivore defense with an ecologically relevant herbivore, the neonate larvae of the *N*. *attenuata* specialist *T*. *mucorea* weevil, which enter the RSJ of *N*. *attenuata* plants to feed on the pith of stems (Fig 3A) and elicit anthocyanin accumulations in the epidermal layers of the RSJ and stems (Fig 3B). We performed larval performance assays with a previously characterized method [11]: to mimic how *T*. *mucorea* attacks plants in nature, freshly collected eggs were inoculated into the petioles of rosette leaves, the natural oviposition sites of *T*. *mucorea* adults. Neonate larvae then burrow through the petioles into the pith of the RSJ, where they feed endophytically inside stems. At 2 or 3 weeks postinoculation (wpi), when larvae in wild-type (WT) stems are typically in the penultimate instar [44], stems were carefully split open, larvae were retrieved, and larval biomass was quantified. To first investigate the direct role of JAs in defense against *T*. *mucorea*, a transgenic line deficient in JA signaling, resulting from the silencing the JA biosynthetic gene, *allene oxide cyclase* (*aoc*) plants [45], was used for the larval performance assay. In contrast to EV plants, *aoc* plants did not show any red-stem phenotype after *T*. *mucorea* attack (Fig 3B), and the larvae performed much better in *aoc* plants (Fig 3C), indicating that JA positively regulates stem anthocyanin accumulation and defense against *T*. *mucorea* attack, as previously reported [11].

To evaluate the role of MAX2 in defense against *T*. *mucorea*, we measured the growth and timing of instar transitions of larvae feeding in stems of *max2* plants. Larvae were significantly larger when feeding on *max2* (#1 and #2) plants than on EV plants (Fig 3D). Furthermore, *T*. *mucorea* larvae consistently performed better in *max2*#2 plants than in EV plants regardless of the feeding period (approximately 1–3 wpi) (Fig 3D, S4A Fig), which implied constitutive (rather than induced) differences between EV and *max2* plants in herbivore resistance. After insect infestation, both EV and *max2* plants showed no differences in stem diameter, plant height, and numbers of primary branches compared with those of uninfested plants (S4B Fig). These results, consistent with field observations [44], suggest that *T*. *mucorea* attack does not morphologically alter plant structures. In addition, larvae were also found most frequently (approximately 80%) feeding on the pith of *max2* stems in larvae choice assays (S4C Fig). Considering the involvement of MAX2 in both SL and KAR signaling pathways, *d14*, *ccd7*, and *kai2* plants were additionally used in feeding assays to more precisely dissect the effects of SL and KAR signaling on herbivore resistance. The larvae feeding in the stems of both *d14* and *ccd7* plants were heavier than those feeding in the stems of EV plants (Fig 3E and 3F), demonstrating that SL signaling is positively correlated with larval performance. However, the performance of larvae feeding in EV or *kai2* plants did not differ significantly (Fig 3G), indicating that KAR signaling is not involved in defensive responses against *T*. *mucorea*.

We identified SL-mediated phytohormonal responses to *T*. *mucorea* attack and changes in SL-reporter genes and SL levels in EV plants. In response to larval feeding, transcript abundances of *BRC1a* and *CCD8* were significantly reduced to about 25% of those in unattacked EV plants, and the levels of (±)-2′-*epi*-orobanchol in roots decreased by 50% after attack (Fig 3H), indicating that *T*. *mucorea* attack leads to the down-regulation of SL responses. JA contents were dramatically elevated in an AOC-dependent manner upon *T*. *mucorea* attack (Fig 3I). In addition to the constitutive responses of IAA and JAs of *max2* plants (Fig 2C), the inducibilities of JA (rather than JA-Ile) and IAA levels in the stems of *max2* and *ccd7* plants in response to *T*. *mucorea* feeding were also consistently higher than those of EV plants (Fig 3J).

To comprehensively illuminate the mechanisms responsible for the attenuated resistance of SL-insensitive plants to *T*. *mucorea*, we conducted a microarray assay to compare constitutive transcriptional changes in the pith of the RSJs of EV and *max2* plants. Multidimensional scaling (MDS) analysis grouped *max2*#1 and *max2*#2 together and clearly separated them from the EV samples (S5A Fig). Differentially expressed genes (DEGs) included 1,160 up-regulated DEGs and 435 down-regulated DEGs (|log2FC| ≥ 1, false discovery rate (FDR) ≤ 0.05) (S5B and S5C Fig), and these were further used to compute the enrichment of Gene Ontology (GO) terms. The GO terms were highly enriched in processes associated with the regulation of meristem development, signaling, hormone metabolism, and transport. Among these, phenylpropanoid metabolism, response to wounding, and auxin transport and response have previously been reported to be involved in plant defense (S5D Fig) [2,5]. Consistently, transcript levels of the wounding-associated genes *NaOPR2a*, *NaOPR2b*, and *JASMONIC ACID RESISTANT 4* (*NaJAR4*) and auxin-related genes of *NaPIN7*, *NaPIN6*, *NaPIN1*, *YUCCA 6* (*NaYUC6*), and *AUXIN RESISTANT 1* (*NaAUX1*) were up-regulated in *max2* samples (S5E Fig). The changes of these genes were consistently observed in the RSJs of *d14* and *ccd7* plants, but not in those of *kai2* plants (S5F Fig). To summarize, SL-RNAi plants are more susceptible to *T*. *mucorea* attack and display amplified JAs and IAA responses in both transcriptional and metabolic layers, regardless of herbivore attack. Given the commonly held knowledge that plant defenses are positively regulated by JA signaling, this apparent disconnect was particularly intriguing.

### Untargeted metabolic profiling revealed changes in specific sectors of specialized metabolism in the RSJs of *max2* plants

**Fig 4:**
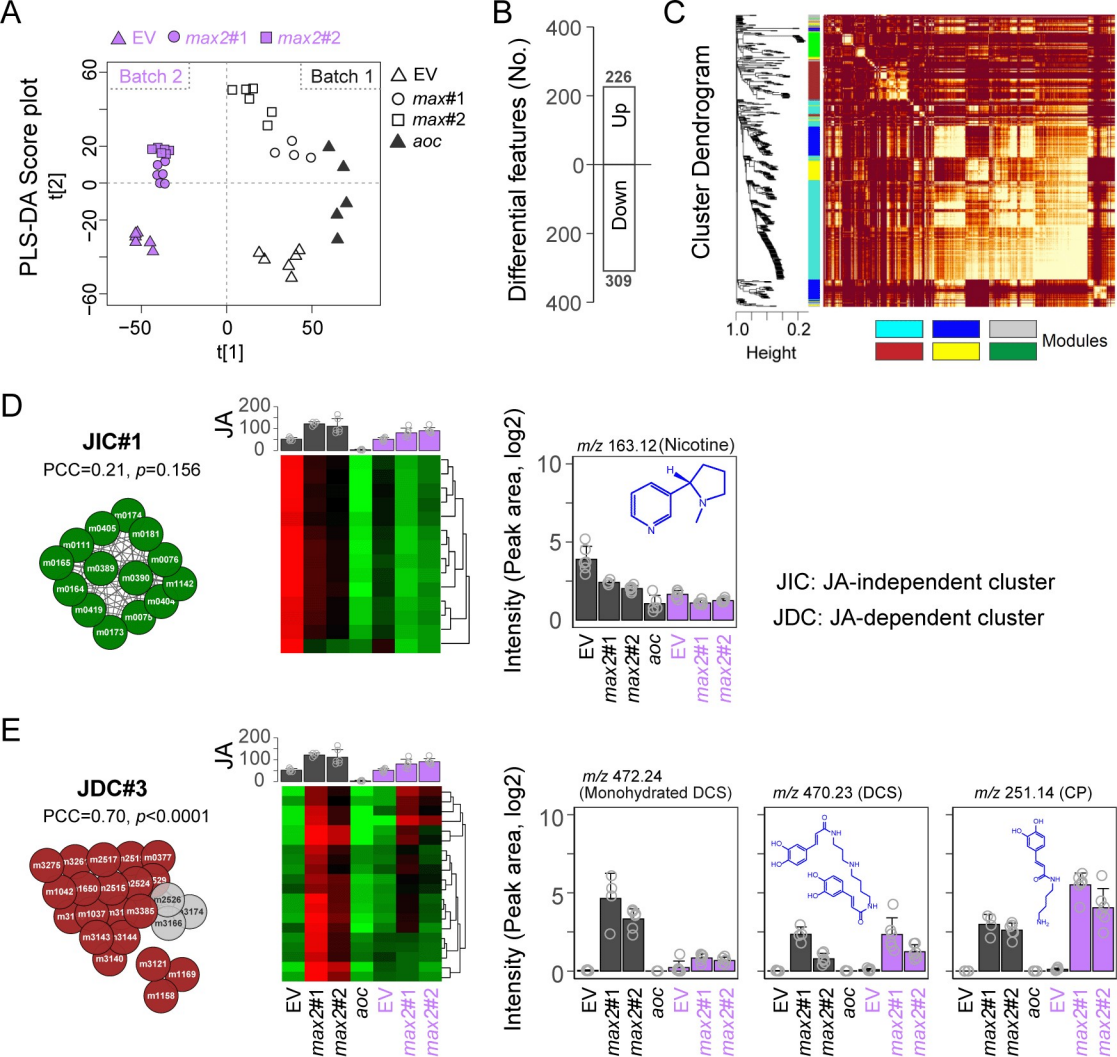
Untargeted metabolic profiling reveals the JA regulation of specific sectors of specialized metabolism in the RSJ of *max2* plants with JA-deficient *aoc* plants as reference. (A) PLS-DA of metabolomes of EV, *aoc*, and *max2* plants in two independent experiments (batch 1 and 2). (B) Number of up-regulated and down-regulated features in total after statistical filtering. (C) TOM plot for visualizing coexpression outcome by WGCNA. (D and E) Extracted subnetworks visualized by Cytoscape (left panels) and correlations with JA contents with each subnetwork were calculated as PCC and their *P* values. Bar charts display the representative compounds from each subnetwork across all samples from two batches (dark gray: first batch; purple: second batch). Values for graphs (D) and (E) are listed in S1 Table. aoc, allene oxide cyclase; CP, *N*′-caffeoylputrescine; DCS, *N*′, *N″*-decaffeospermidine; EV, empty vector; JA, jasmonate; JDC, JA-dependent cluster; JIC, JA-independent cluster; max2, more axillary growth 2; PCC, Pearson’s correlation coefficient; PLS-DA, partial least squares discriminant analysis; RSJ, root–shoot junction; TOM, topological overlap matrix; WGCNA, weighted correlation network analysis.

The apparent disconnect between the observed higher JA/JA-Ile levels and the greater susceptibility to herbivore attack in *max2*, *d14*, and *ccd7* plants led to the prediction that particular sectors of defense metabolism in SL-RNAi plants were not primarily regulated by JA signaling. To better understand the underlying metabolic basis of resistance to *T*. *mucorea* attack, two batches of RSJ samples from EV, *max2*#1, *max2*#2, and *aoc* plants (41 in total) were analyzed by untargeted metabolome profiling using liquid chromatography (LC) coupled to quadrupole time-of-flight mass spectrometry (qTOF-MS). This analysis resulted in a concatenated data matrix consisting of 5,975 mass features (*m/z* signals detected at particular retention times). A supervised principal components analysis using partial least squares discriminant analysis (PLS-DA) clearly separated the *max2* samples, which nicely grouped together (Fig 4A). For each batch, Student *t* tests with Benjamin–Hochberg corrections among comparisons with EV samples were performed. This analysis identified 226 up-regulated and 309 down-regulated mass features, which were further used for coexpression analyses with the following significance requirements: |log2FC| > 0.58, FDR < 0.05 (Fig 4B, S1 Table).

A well-developed coexpression pipeline, the weighted correlation network analysis (WGCNA) [46], was employed, and six modules were obtained (power = 10) (Fig 4C). Samples of the RSJs of *aoc* plants were used to identify JA/JA-Ile–independent/–dependent subnetworks. Based on this, coexpression networks were constructed (topological overlap matrix [TOM] > 0.3) (Fig 4D and 4E and S6 Fig), and JA-independent clusters (JICs)/JA-dependent clusters (JDCs) were defined depending on whether subnetworks were significantly correlated with the JA/JA-Ile contents of the samples. As indicated in S6 Fig, *O*-acyl sugars were nicely clustered in the blue module and tightly associated with a similar enrichment pattern across all groups, revealing the robustness of network construction. However, in this large subnetwork, variable expression patterns correlated with JA/JA-Ile contents. In the cluster JIC#1, all features decreased consistently in *max2* and *aoc* groups regardless of JA/JA-Ile contents (*P* = 0.156) (Fig 4D). Interestingly, in JIC#1 cluster annotated as alkaloids, we found the levels of nicotine to be significantly decreased in *max2* and *aoc* samples (Fig 4D). On the other hand, we found compounds with significant negative correlations with JA/JA-Ile contents in unknown clusters JDC#1 (PCC = −0.41, *P* = 0.021) (S6B Fig) and JDC#2 (PCC = −0.76, *P* < 0.0001) (S6C Fig) and compounds with positive correlations with JA/JA-Ile contents in JDC#3 (PCC = 0.70, *P* < 0.0001) (Fig 4E), which were mainly populated by known phenolamide structures. After these analyses, the associated features in the largest subnetwork from JIC#1 and JDC#3 were captured for further analysis.

### Increased phenolamide accumulations in SL-RNAi RSJ are positively related to JA signaling

**Fig 5:**
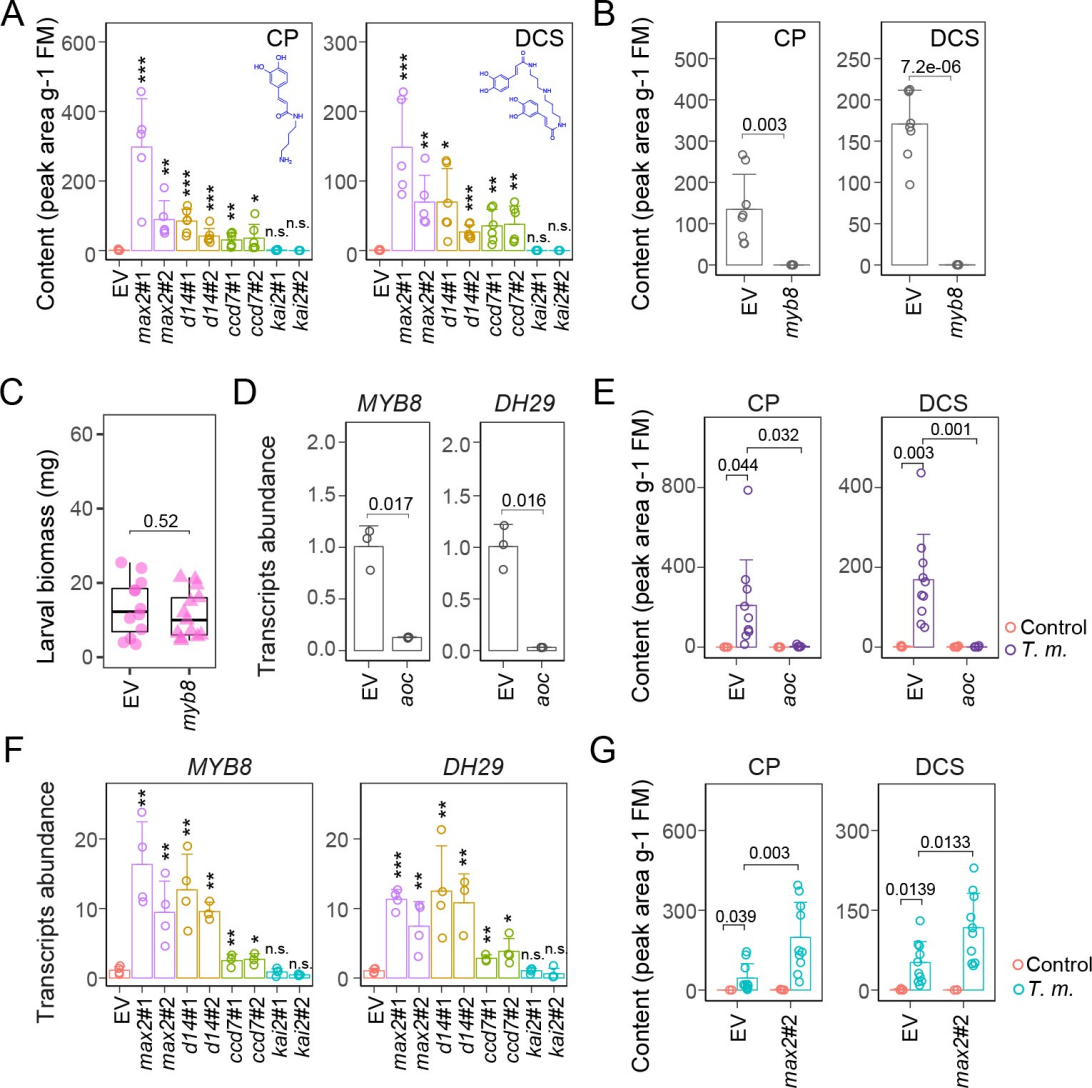
Increased phenolamide accumulations in SL-RNAi RSJ are positively related to JA signaling via *MYB8*. (A) Relative contents of phenolamides such as CP and DCS in the RSJs of indicated plants (±SE, *n =* 6). (B) Relative contents of CP and DCS in RSJs of EV and *myb8* plants after attack. (C) Biomass of *T*. *m*. larvae after attacking EV and *myb8* plants for 2 weeks (*n =* 15). (D) Relative transcript abundance of *MYB8* and *DH29* in RSJ of EV and *aoc* plants (±SE, *n =* 4). (E) Relative contents of CP and DCS in the pith of EV and *aoc* plants without (Control) or with *T*. *m*. attack for 2 weeks. (F) Relative transcript abundance of *MYB8* and *DH29* in the RSJs of indicated plants (±SE, *n =* 4). (G) Relative content of CP and DCS in the pith of EV and *max2*#2 plants without (Control) or with *T*. *m*. attack for 2 weeks (±SE, *n =* 6–10) (*P < 0.05; **P < 0.01; ***P < 0.001; two-tailed Student *t* test). Values for graphs (A–G) are listed in S1 Data. aoc, allene oxide cyclase; ccd, carotenoid cleavage dioxygenase; CP, *N*′-caffeoylputrescine; d14, dwarf14; DCS, *N*′, *N″*-decaffeospermidine; *DH29*, *ACYLTRANSFERASE DH29*; EV, empty vector; JA, jasmonate; kai2, karrikin insensitive 2; max2, more axillary growth 2; n.s., not significant; RNAi, RNA interference; RSJ, root–shoot junction; SL, strigolactone; *T*. *m*., *T*. *mucorea*.

Levels of phenolamides located in cluster JDC#3 were increased in the RSJs of *max2* plants, which is consistent with the presence of significant GO enrichment in phenylpropanoid biosynthetic genes (S5D Fig). Importantly, the signature compounds of phenolamides, *N*′-caffeoylputrescine (CP) and *N*′, *N″*-decaffeospermidine (DCS), were also increased in *ccd7* and *d14* plants, but not in *kai2* plants (Fig 5A). Because phenolamides are known to play essential roles in defense against leaf feeding lepidopteran herbivores [12,13,47], we performed a *T*. *mucorea* larval performance assay with a transgenic line silenced in *MYB DOMAIN PROTEIN 8* (*MYB8*) expression, a key transcriptional factor regulating phenolamide biosynthesis in *N*. *attenuata* [13], to clarify whether increased CP and DCS in pith of SL-RNAi plants contribute to altered resistance against the stem-boring weevil. However, even though the levels of CP and DCS were much lower in the RSJs of *myb8* plants (Fig 5B), larval performance in *mby8* plants was similar to that in EV plants (Fig 5C). These results indicate that phenolamides do not function as defenses against *T*. *mucorea* larvae.

Consistent with previous findings in leaves [47], transcript abundances of key genes in phenolamide biosynthesis, including *MYB8* and *acyltransferase DH29* (*DH29*), were dramatically lower in the pith of *aoc* plants (Fig 5D); and the increases in CP and DCS levels after *T*. *mucorea* attack were completely lacking in *aoc* plants (Fig 5E), suggesting that the decreased JA/JA-Ile levels lower the production of phenolamides by down-regulating the expression of *MYB8* and *DH29* genes. In SL-insensitive and SL-deficient *max2*, *d14*, and *ccd7* lines, the transcript abundances of *MYB8* and *DH29* were up-regulated in the pith (Fig 5F). CP and DCS levels were also higher in the pith of *max2* plants compared with those of EV plants after *T*. *mucorea* attack (Fig 5G). Taken together, the elevated JA levels may account for the increases in phenolamides via the up-regulation *MYB8* and *DH29* in SL-RNAi lines; this inference allowed us to disentangle the potential cross talk among SL and JA signaling pathways through the crossing of different RNAi lines that targeted key genes in these signaling pathways.

### Phenolamide and anthocyanin accumulations are regulated by SL signaling via JA signaling

**Fig 6:**
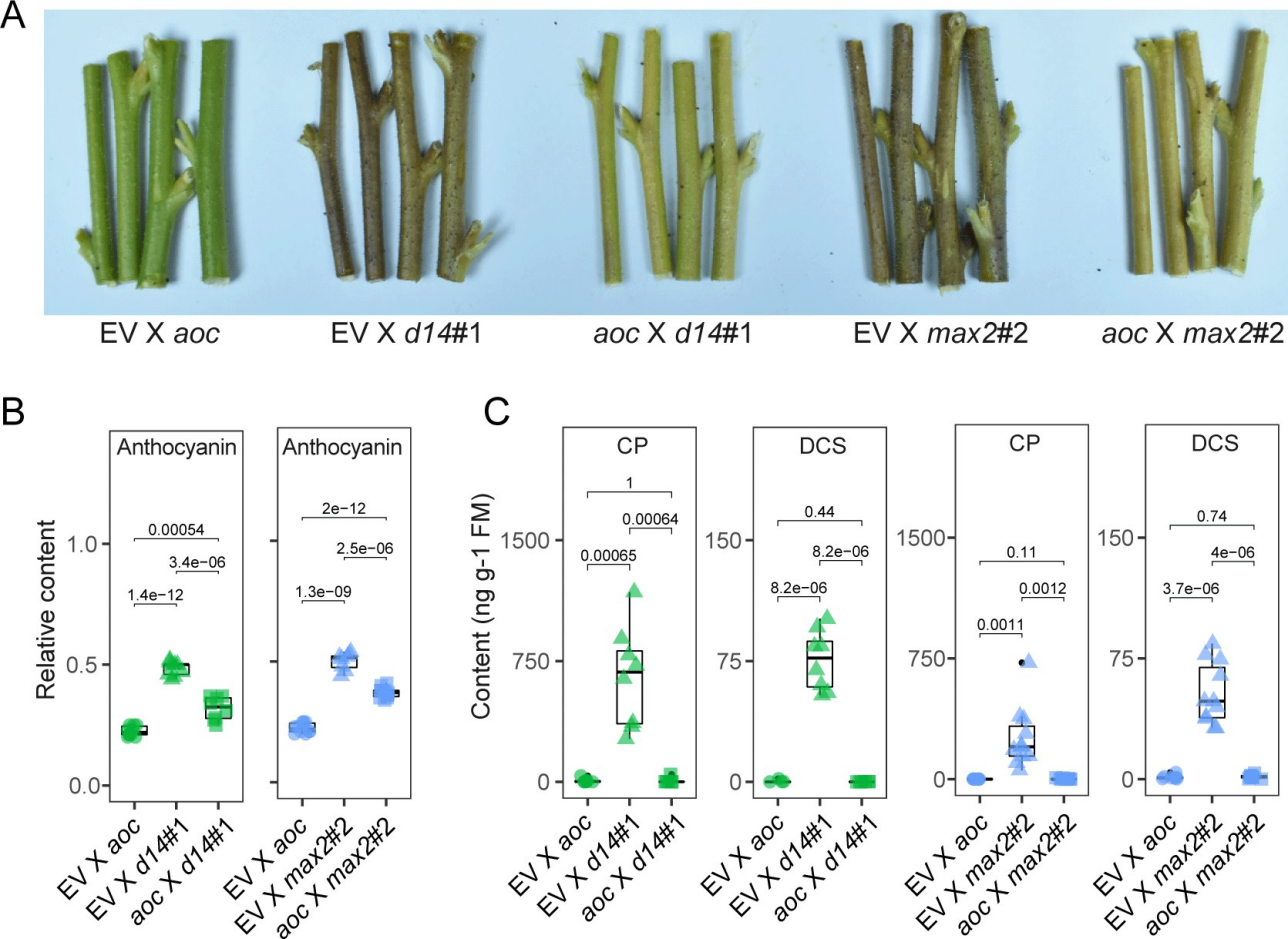
Phenolamide and anthocyanin accumulations are regulated by SL signaling via JA signaling. (A) Representative pictures of stems of EV, EV×*d14*#1, *aoc*×*d14*#1, EV×*max2*#2, and *aoc*×*max2*#2 plants. (B) Relative anthocyanin levels in epidermis of stems of indicated plants (*n =* 8). (C) Relative contents of CP and DCS in stem pith of indicated plants after 2 weeks of *T*. *mucorea* attack (*n =* 8) (two-tailed Student *t* test). Values for graphs (B) and (C) are listed in S1 Data. aoc, allene oxide cyclase; CP, *N*′-caffeoylputrescine; d14, dwarf 14; DCS, *N*′, *N″*-decaffeospermidine; EV, empty vector; max2, more axillary growth 2; JA, jasmonate; SL, strigolactone.

As shown in Figs. 2 and 5, anthocyanin, phenolamides, and JAs all overaccumulate in the stems of SL-RNAi plants, whereas in *aoc* plants, extremely low JA levels are associated with low anthocyanin and phenolamide levels (Fig 3B, Fig 5E). To further examine this interplay of JA-SL signaling, a set of genetic hybridizations were designed. *d14* and *max2* plants with their high JA levels were crossed with the JA-deficient *aoc* plant. We quantified anthocyanin and phenolamide levels in all crosses. The anthocyanin levels in the stems of EV×*d14* and EV×*max2* plants were significantly higher than those in *aoc*×*d14* and *aoc*×*max2* plants, a result consistent with the higher JA levels in both EV×*d14* and EV×*max2* plants (Fig 6A and 6B) and clearly demonstrating that JA signaling functions downstream of SLs to regulate anthocyanin and phenolamide accumulations. Notably, although the increases in anthocyanin levels in *d14* and *max2* stems were attenuated in crosses with *aoc* plants, the levels of anthocyanins in *aoc*×*d14* and *aoc*×*max2* stems were still significantly higher than those in EV×*aoc* stems (Fig 6A and 6B), suggesting that another additional factor regulates anthocyanin accumulations. As auxin is known to regulate anthocyanin levels in *N*. *attenuata* [5] and occurs in higher concentrations in the stems of SL-RNAi plants, we inferred that auxin would positively contribute to the anthocyanin accumulations in SL-RNAi stems. In contrast to the results that both JA and IAA likely regulate anthocyanin accumulation, SL regulates phenolamide production primarily through JA signaling, as the phenolamide contents were almost undetectable in EV×*aoc*, *aoc*×*d14*, and *aoc*×*max2* piths, whereas the levels of phenolamides were dramatically increased in EV×*d14* and EV×*max2* piths after *T*. *mucorea* attack (Fig 6C). Taken together, by crossing SL- and JA-RNAi plants, we dissected the functional consequences of accumulated JA levels in SL-insensitive plants to reveal that the concentrations of anthocyanin and phenolamides in stems of SL-insensitive plants increase in a JA-dependent manner. This insight motivated a deeper exploration of the molecular mechanism involved in this cross talk.

### Interplay of SL and JA signaling through SMXL6/7-JAZ interactions

**Fig 7:**
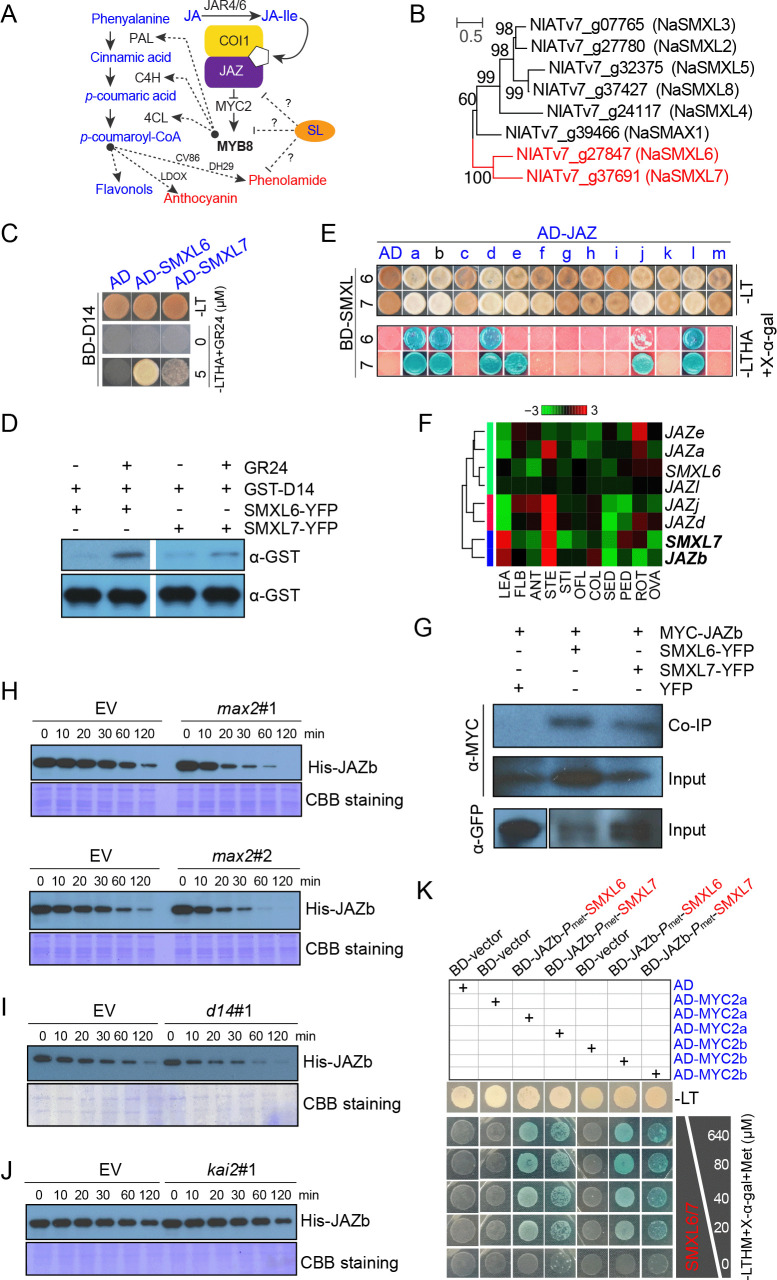
Interplay of SL and JA signaling through SMXL6/7-JAZ interactions. (A) Schematic diagram of amplified JA signaling resulting from silenced SL signaling that leads to accumulations of anthocyanins and phenolamides. (B) Phylogenetic analysis of *NaSMXL* gene family from *N*. *attenuata*. (C) Interactions between NaSMXL6/7 and NaD14 proteins in the presence of *rac*-GR24 by Y2H assays. GAL4 DNA-BD-D14 and AD-SMXL6/7 were cotransformed into yeast. The transformants were grown on QDO (SD−Ade/−His/−Leu/−Trp). (D) The interactions of NaSMXL6 /7 and NaD14 in the presence of 20 μM *rac*-GR24 by pull-down assays. NaSMXL6/7-YFP was transiently expressed in *N*. *benthamiana* leaves. Purified GST-D14 was used. Input of GST-D14 is shown at the second panel. (E) Interaction between NaSMXL6/7 and NaJAZs proteins by Y2H assays. GAL4 DNA-BD-SMXL6/7 and AD-JAZs were cotransformed into yeast. The transformants were grown on QDO (SD −Ade/−His/−Leu/−Trp with 40 μg/mL X-α-gal). (F) Relative transcript abundance of *SMXL6*, *SMXL7*, and those *JAZs* that encode JAZ proteins that interact with SMXL in different tissues. Expression levels were analyzed from microarray data. (G) Interactions of SMXL6/7-YFP and MYC-JAZb by in vivo Co-IP. (H) JAZb degradation in EV and *max2* (#1, #2) crude proteins. Purified His-JAZb was incubated in EV or *max2* crude proteins extracted from stem pith for the indicated times. His-JAZb was detected by anti-His. The CBB staining represented protein loading levels. (I-J) JAZb degradation in EV, *d14*#1, or *kai2*#1 crude proteins, with the same condition mentioned in (H). (K) Interference of SMXL6/7 on interaction of JAZb and MYC2a/b by Y3H assay. The expression of SMXL6/7 was gradually induced by the addition of decreasing amounts of Met. The transformants were grown on QDO (SD−Leu/−His/−Trp/−Met with 40 μg/mL X-α-gal). Raw images for blots are listed in S1 Raw Images. AD, activation domain; ANT, anther; BD, binding domain; CBB, Coomassie Brilliant Blue; COI1, coronatine insensitive 1; Co-IP, co-immunoprecipitation; COL, corolla late; d14, dwarf 14; EV, empty vector; FLB, flower bud; GST, glutathione S-transferase; JA, jasmonate; JAR4/6, JASMONIC ACID RESISTANT 4/6; JAZ, JASMONATE ZIM-DOMAIN; kai2, karrikin insensitive 2; *LDOX*, *LEUCOANTHOCYANIDIN DIOXYGENASE*; LEA, leaf; LT, Leu and Trp; LTHA, Leu, Trp, His, and Ade; max2, more axillary growth 2; Met, methionine; MYB8, MYB DOMAIN PROTEIN 8; OFL, opening flower; OVA, ovary; PED, pedicel; QDO, quadruple dropout medium; ROT, root; SD, synthetic defined medium; SED, seed; SL, strigolactone; SMXL, suppressor of max2-like; STE, stem; STI, stigma; Y2H, yeast two-hybrid; Y3H, yeast three-hybrid; YFP, yellow fluorescent protein.

Based on the crossing results, we inferred that SL tunes the responses of the JA signaling pathway through an unknown mechanism to regulate anthocyanin and phenolamide production through COI1/JAZ and transcriptional factors such as MYB8 (Fig 7A). Given that the direct interaction of JAZs with DELLA proteins contributes to the cross talk between JA and GA signaling [48], we hypothesized that physical interactions among the suppressors of SL and JA signaling could account for the interplay between SL and JA signaling (Fig 7A). After BLAST *N*. *attenuata* genome searches with published sequences of suppressors of MAX2, SMXL6/7/8 from *Arabidopsis*, two orthologues (NaSMXL6 and NaSMXL7) were identified (Fig 7B and S7A Fig). NaSMXLs were localized to the nucleus and found to directly interact with NaD14 in the presence of the synthetic SL analogue *rac*-GR24 (5 μM) (Fig 7C and 7D, S7B and S7C Fig).

To test whether the SL repressors, NaSMXLs, interact with the JA repressors, NaJAZs, we conducted yeast two-hybrid (Y2H) assays. The results suggested that both SMXL6 and SMXL7 interacted with JAZa, JAZb, JAZd, JAZj, and JAZl, whereas JAZe only interacted with SMXL7 (Fig 7E). Coexpression analysis was performed using RNA-seq data across different tissues [49] to identify patterns among genes, including SMXLs and the JAZs (a/b/d/j/l/e). The stem-abundant SMXL7 was found to be highly coexpressed with JAZb (Fig 7F), and JAZb was used for further studies. We further confirmed the interaction of SMXL6/7 and JAZb in vivo using coimmunoprecipitation (Co-IP) assays. MYC-JAZb physically interacted with SMXL6/7-YFP when transiently expressed in *N*. *benthamiana* leaves (Fig 7G).

In *max2* plants, SMXL6 protein was found to accumulate to higher levels than in EV plants, as determined by immunoblots with anti-SMXL6 (S7D Fig). According to the degradation mechanisms elucidated in *Arabidopsis* and rice [26,50], a greater accumulation of SMXL7 was predicted; however, because of a lack of antibody against SMXL7, this was not determined. To determine whether larger accumulations of SMXL6/7 proteins in *max2* plants influence JAZb degradation, we performed an in vitro degradation assay by adding recombinant His-JAZb to crude protein extracted from the pith of EV or *max2* plants and quantified the degradation of JAZb after incubation for the indicated time points. His-JAZb degraded more rapidly in *max2* crude protein relative to that in EV (Fig 7H). As reported in rice, more D53 protein was found to accumulate in both *d14* and *max2* plants [50]; hence, we performed degradation assays with *d14* crude protein. As expected, JAZb was degraded more rapidly in *d14* crude protein extracts (Fig 7I). Because of the close phylogenetic relationships between D14 and KAI2, *kai2* lines were also included in the tests of the stability of the His-JAZb complex. The pattern of His-JAZb degradation in *kai2* crude protein extracts was found to be similar to its degradation in EV crude protein extracts (Fig 7J). In contrast to the more rapid degradation of JAZb when interacting with SMXL6/7, His-JAZk, one of the JAZs not interacting with SMXL6/7 in the Y2H assays, degraded at a similar rate in EV and *max2* crude protein extracts (S7E Fig).

Additional Y3H assays were performed to explore the effect of SMXL6/7 on the interaction of JAZb and MYC2a/b. With decreasing methionine (Met) concentrations in the media, the abundance of SMXL6 or SMXL7 was experimentally increased, and the binding capacity of JAZb-MYC2 was gradually weakened until completely abolished, when no Met was added to the media (Fig 7K and S7F Fig). This result indicates that overaccumulated SMXL6/7 dampens the binding between MYC2 and JAZb.

In summary, the enhanced SMXL6/7 protein in SL-RNAi plants physically interacts with JAZs to trigger the degradation of JAZ protein; excessive SMXL6/7 protein production interferes with the interactions of JAZs and MYC2, which both facilitate the release of MYC2 to activate the downstream JA signaling cascade, which results in enhanced anthocyanin and phenolamide accumulations in SL-RNAi plants.

### Decreased nicotine mediated by increased auxin accounts for the susceptibility of SL-RNAi plants to *T*. *mucorea* attack

**Fig 8:**
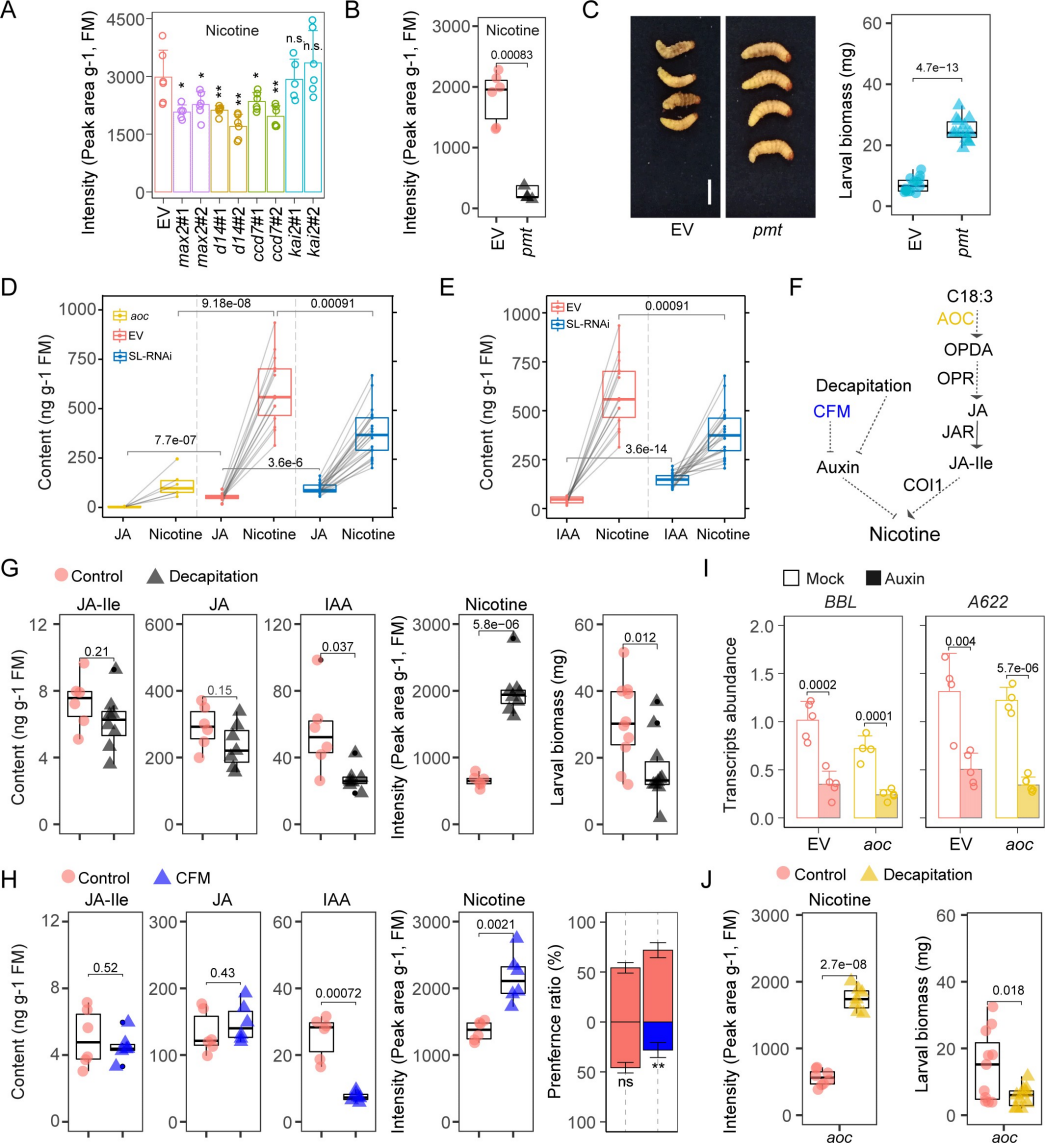
Decreased nicotine mediated by increased auxin accounts for the susceptibility of SL-RNAi plants to *T*. *mucorea* attack. (A) Relative contents of nicotine in the RSJs of indicated plants (±SE, *n =* 6). (B) Relative levels of nicotine in the pith of EV and *pmt* plants (*n =* 6). (C) Representative pictures and biomass of *T*. *mucorea* larvae after attacking EV and *pmt* plants for 2 weeks (*n =* 15). Scale bar, 2 mm. (D) Relationship of JA and nicotine levels in RSJ of *aoc*, EV, and SL-RNAi (*max2*, *d14*, and *ccd7*) plants. Data collected from same samples are indicated by gray lines. (E) Relationship of IAA and nicotine levels in RSJ of EV and SL-RNAi (*max2*, *d14*, and *ccd7*) plants. Data collected from the same sample are connected with a gray line. (F) Schematic diagram of nicotine biosynthesis regulated by auxin and JA-Ile. (G) Levels of JA-Ile, JA, and IAA in roots of EV plants without (Control) or with decapitation treatment for 3 days (*n =* 6–8). Nicotine content in the RSJs of EV plants with or without decapitation treatments for 3 days (*n =* 5–6). Biomass of larvae that had attacked intact or decapitated EV plants for 2 weeks (*n =* 12). (H) Levels of JA-Ile, JA, and IAA in roots of EV plants without (Control) or with treatments with the IAA transport inhibitor CFM for 3 days (*n =* 6). Nicotine levels in RSJ of EV without or with CFM treatment for 3 days (*n =* 6). Pith-preference bioassay with *T*. *mucorea* larvae between EV stem halves EV (Control)-EV (Control), EV (Control)-EV (CFM treatment) (±SE, 3 replicates, each replicate includes 8–10 plants). (I) Relative transcript abundance of nicotine biosynthetic genes *BBL* and *A622* in EV and *aoc* seedlings without (Mock) or with 10 μM IAA treatment for 8 hours (±SE, *n =* 4). (J) Nicotine contents in RSJ of *aoc* plants without (Control) or with decapitation treatment (*n =* 6). Biomass of larvae that had attacked *aoc* control plants or decapitated plants for 2 weeks (*n =* 12) (two-tailed Student *t* test). Values for graphs (A–E and G–J) are listed in S1 Data. *A622*, *ISOFLAVONE REDUCTASE-LIKE PROTEIN*; aoc, allene oxide cyclase; *BBL*, *BERBERINE BRIDGE ENZYME-LIKE*; ccd, carotenoid cleavage dioxygenase; CFM, methyl-2-chloro-9-hydroxyfluorene-9-carboxylate; COI1, CORONATINE INSENSITIVE 1; d14, dwarf14; EV, empty vector; IAA, indole-3-acetic acid; JA, jasmonate; JA-Ile, jasmonic acid-isoleucine; *JAR*, *JASMONIC ACID RESISTANT*; kai2, karrikin insensitive 2; max2, more axillary growth 2; n.s., not significant; OPR, OPDA reductase; pmt, putrescine n-methyltransferase; RNAi, RNA interference; RSJ, root–shoot junction; SL, strigolactone.

Even though the enhanced phenolamides do not account for the susceptibility of SL-RNAi plants to *T*. *mucorea* attack (Fig 5B and 5C), we noticed that another essential defense compound in the JIC#1 module, nicotine, was decreased in the RSJs of *max2* samples from the untargeted metabolome analysis Fig 4D. Nicotine levels were consistently lower in the pith of *max2* plants compared with those of EV plants regardless of *T*. *mucorea* infestation (S8A Fig); they were also significantly lower in the roots of *max2* plants after attack (S8B Fig). JA and IAA levels were higher in the roots of *max2* plants (S8C Fig), consistent with their levels in pith (Fig 2C). Further analysis revealed that nicotine levels were significantly decreased in the RSJs of *max2*, *d14*, and *ccd7* plants, but not in those of *kai2* plants (Fig 8A). To evaluate whether nicotine, which is synthesized in roots, is essential for plant defense against *T*. *mucorea* larvae, we performed feeding assays using a nicotine-deficient transgenic line, *putrescine n‐methyltransferase* (*pmt*) plants (Fig 8B) [51]. *T*. *mucorea* larvae inoculated into *pmt* plants gained higher biomass compared with those that fed on EV plants (Fig 8C). It is known that JA signaling stimulates nicotine biosynthesis and accumulation [8,52]; however, in the RSJs of SL-RNAi plants, low nicotine levels coincide with high JA/JA-Ile levels (Fig 8D). Auxin signaling is known to suppress nicotine levels [52,53], which suggests that the high IAA contents cause the low nicotine levels in SL-RNAi plants (Fig 8E).

To test whether the decreased amount of nicotine in *max2* plants resulted from increased IAA levels, we manipulated auxin levels by decapitation or using the auxin transport inhibitor methyl-2-chloro-9-hydroxyfluorene-9-carboxylate (CFM) (Fig 8F). Three days after decapitating EV plants, there were no significant changes in JA and JA-Ile levels, whereas IAA decreased about 2-fold (Fig 8G). The amount of nicotine significantly increased in the RSJs in decapitated EV plants (Fig 8G). Moreover, *T*. *mucorea* larvae performed worse in decapitated EV plants compared with those in intact plants (Fig 8G). Next, after using CFM to block IAA transport from shoot to root, IAA levels decreased in EV plants without altering JA or JA-Ile levels (Fig 8H). The amount of nicotine in the RSJs of EV plants after CFM treatment was higher than in control plants (Fig 8H). In pith-preference assays, more than 70% of the larvae preferred to feed on pith from plants subjected to the CFM treatment than from control pith (Fig 8H). To further evaluate whether auxin regulates nicotine levels independently of JA signaling, we treated *aoc* seedlings with IAA for 8 hours. Transcript levels of the nicotine biosynthetic genes *BBL* and *A622* decreased to similar degrees (approximately 30%) in both EV and *aoc* seedlings (Fig 8I). Furthermore, *aoc* plants still accumulated more nicotine in their RSJs after decapitation, again leading to a lower larval biomass gain (Fig 8J).

To better understand the role of the increased auxin levels in *max2* plants in *T*. *mucorea* resistance, we studied the nicotine biosynthesis response of *max2* plants to auxin. Transcripts of *BBL* and *A622* were lower in *max2*#2 than in EV seedlings (S8D Fig), suggesting that the biosynthesis of nicotine in *max2* plants was decreased. After auxin treatment, the expression of *BBL* and *A622* was still decreased in *max2* plants, as in EV plants (S8D Fig). Upon decapitation, the amount of nicotine was enhanced in the RSJs of *max2*#2 plants, resulting in significantly lower larval performance compared with undecapitated plants (S8E Fig). These results demonstrate that *max2*’s nicotine biosynthesis machinery still responds to changes in auxin levels.

In summary, these results reveal that the inhibitory effect of auxin on nicotine accumulation is at least partially independent of JA signaling, and it is the change in nicotine levels associated with SL silencing that most profoundly determine larval performance.

### SL signaling balances the metabolic output of specialized sectors via its interaction with JA and auxin

**Fig 9:**
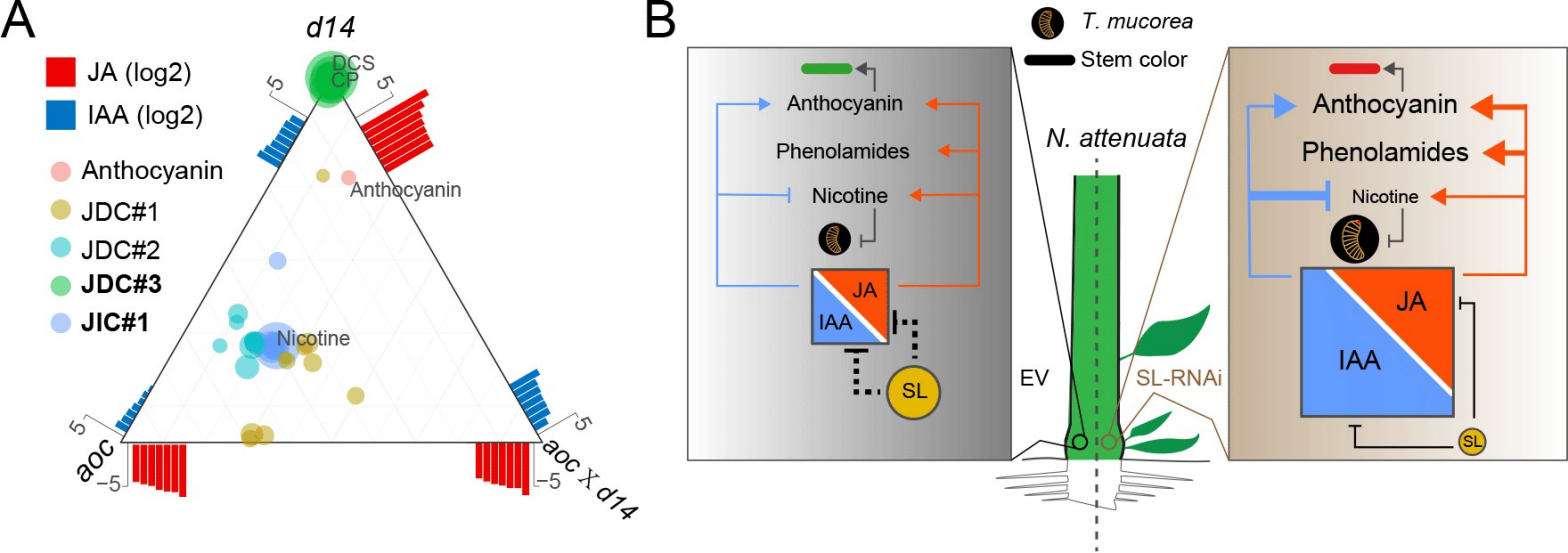
SL signaling regulates anthocyanin accumulations and plant defense against *T*. *mucorea* larval attack via its interaction with JA and auxin signaling. (A) A ternary analysis illustrating metabolite distributions across three genotypes: *aoc*, *d14*, and *aoc*×*d14* plants. For JIC#1 and JDC#1–3, see Fig 4 and S6 Fig. (B) A model of the role of SLs as regulators that fine-tune resistance to endophytic stem-feeding weevils by disrupting the SL-mediated homeostasis between auxin and JA signaling. In *N*. *attenuata*, SL-deficient (*d14*, *max2*, and *ccd7*) plants produce more branches (right panel) relative to EV (left panel), have lower pith nicotine levels and higher anthocyanin and phenolamine levels, and are more susceptible to *T*. *mucorea* attack. In EV plants, SL levels maintain auxin and JA homeostasis, but once SL signaling is impaired, auxin and JA levels increase and become unbalanced, decreasing pith nicotine levels and increasing susceptibility to larval attack. Values for the graph (A) are listed in S1 Data. aoc, allene oxide cyclase; CP, *N*′-caffeoylputrescine; d14, dwarf14; DCS, *N*′, *N″*-decaffeospermidine; EV, empty vector; JA, jasmonate; IAA, indole-3-acetic acid; JDC, JA-dependent cluster; JIC, JA-independent cluster; max2, more axillary growth 2; SL, strigolactone.

We returned to the JDC and JIC metabolite modules to understand the ternary regulatory relationship dissected by the *d14*, *aoc*, and *d14×aoc* lines. As previously mentioned, the pith of *d14* displayed increased auxin and JA levels, *aoc* pith had lower JA but normal auxin levels, and *d14×aoc* pith exhibited higher auxin but lower JA levels. As indicated in Fig 4E, JDC#3 (phenolamide sector) was dominantly correlated with JA levels, and anthocyanin production was also mainly correlated with JA levels, although IAA was also involved in this regulation (Fig 9A). The JIC#1 module (alkaloid sector) included nicotine and clustered with *aoc* largely because of the inhibition effect of higher auxin levels from the *d14* background. The JDC#1 and JDC#2 modules negatively correlated with JA levels (S6B and S6C Fig) and clustered distinctly because of the larger repression effect of higher JA levels in *d14* plants (Fig 9A). This analysis clearly illustrated that the metabolic profiles of the pith reflect the balance amongst several regulators, and this balance is tuned by additional factors such as SL signaling.

As summarized in the proposed model (Fig 9B), the RSJs of SL-RNAi plants accumulate SL repressors SMXL6 and SMXL7, which directly interact with JAZs, accelerating JAZ degradation, releasing the inhibition of MYC2 from JAZ to amplify JA signaling, and increasing the production of phenolamides and anthocyanin through MYB8-mediated regulation. Meanwhile, auxin may contribute to the anthocyanin production as well [5]. However, these increases in defense responses do not attenuate the growth of the stem-boring larvae. Even though SL-RNAi lines (*max2/d14/ccd7*) exhibited enhanced JA responses, which positively regulated nicotine biosynthesis, the simultaneous increases in IAA levels completely neutralized JA’s effects to decrease nicotine levels in the pith, which accounted for the greater larval performance.

## Discussion

A new class of phytohormones, SLs, and their cross talk with other hormones have been intensively studied for their function in regulating plant development, stimulating parasitic seed germination, and associating with arbuscular mycorrhizal fungi (AMF); however, their function in regulating herbivore defense was unknown. In this study, we demonstrate that SL signaling, but not KAR signaling, plays an essential role in resistance against a native endophytic pith-feeding herbivore, *T*. *mucorea*, through a complex, functionally important phytohormonal cross talk with JAs and auxin. The motivation for exploring this cross talk was the novel observation that all SL-RNAi lines in *N*. *attenuata*, but not the *kai2*-RNAi line, displayed a dramatic red-stem phenotype that resulted from the accumulation of anthocyanins (Fig 2A and 2B). Anthocyanins have been implicated in plant tolerance to abiotic and biotic stressors, and their biosynthesis is controlled by a variety of stresses, such as UV, cold, and herbivore attack [43]. In *N*. *attenuata*, this novel discovery, which was not reported for the better-studied mutants in *Arabidopsis* or rice [16], provided the link to herbivore resistance; when we hunt for plants in native populations in the field that clandestinely harbor these endophytic weevils in their stems, the most reliable marker is the red pigmentation that anthocyanin imparts to stems. In the glasshouse, stems turn red approximately 1 week after *T*. *mucorea* eggs are inoculated into stems when these inoculations result in successful larval infestations of WT, but not JA-deficient plants (Fig 3B). The functional importance, if any, of this red pigmentation response remains to be explored in future field research. Red stems may benefit the endophytic weevils by raising stem temperatures, speeding development, and/or changing the frass microbiomes that likely facilitate the weevil’s coprophagic behavior inside stems [54]. Alternatively, the red pigmentation may function as a visual indirect defense, functionally analogous to the volatile alarm calls that are so effective in attracting predators to herbivore-attacked *N*. *attenuata* plants [55]. In contrast, the functional significance of nicotine and other changes in pith secondary metabolism for weevil performance was characterized in this and previous publications [11,41,44], which provide insights into how natural selection has sculpted a skein of direct and indirect phytohormonal responses to attack from this native herbivore whose life is intimately entwined with that of its host.

Nicotine is an abundant pyridine alkaloid in many *Nicotiana* species and an efficient neurotoxin that poisons unadapted herbivores and retards the growth of nicotine-tolerant herbivores [51,56]. Two species of *Trichobaris*, *T*. *compacta* and *T*. *mucorea*, which attack tobacco, tomato, or other solanaceous plants such as *Datura wrightii*, both infest *N*. *attenuata* in their native habitat [44]. The larvae of these weevils spend their entire premating lives (approximately 4 months) inside the stems of plants until they metamorphose to adults when the plants senesce [44]. Larvae of both species can survive in the stems of *D*. *wrightii*, which accumulates the alkaloid scopolamine but produces no nicotine, whereas only *T*. *mucorea* is sufficiently nicotine tolerant to survive in *N*. *attenuata* stems [44]. High levels of pith nicotine slow the growth of *T*. *mucorea* larvae rather than kill them, which is similar to the effect of nicotine on the famously nicotine-resistant *Manduca sexta* larval folivore [57].

Here, we show that *T*. *mucorea* larvae grow larger in nicotine-deficient *pmt* plants (Fig 8C), in agreement with the previous report that *T*. *mucorea* larvae consistently choose to feed on the pith of *pmt* plants over EV plants in split-stem pith-preference bioassays [46] and also consistent with the inference that nicotine plays a central role in the susceptibility of SL-RNAi plants. In all SL-RNAi plants, nicotine levels were significantly lower compared with those in EV plants (Fig 8A), though with slightly different levels among the different lines, which in turn may reflect the minor differences in silencing efficiency of the target genes (Fig 1C). Nicotine is synthesized in roots and transported to aboveground parts [9]. As nicotine levels in the pith are determined not only by transport from its site of synthesis in the roots but also from reallocation processes that move nicotine into different shoot tissues, it is difficult to clearly evaluate the reasons for the lower nicotine levels in the pith of *max2*, *d14*, and *ccd7* plants based on the available data. Even though the constitutive root nicotine levels do not appear to differ significantly between EV and *max2* plants (S8B Fig), the transcript levels of nicotine biosynthetic genes *BBL* and *A622* were significantly lower in *max2* than in EV seedlings (S8D Fig), indicating decreased nicotine biosynthesis in *max2* plants. Moreover, in contrast to the induced nicotine amounts in EV roots after *T*. *mucorea* attack, nicotine levels in *max2* roots were significantly lower than those in EV roots in response to infestation (S8B Fig), indicating a positive role of SL signaling in eliciting nicotine biosynthesis in response to herbivore attack. On the other hand, pith nicotine levels were decreased by attack (S8A Fig), and we infer that destruction of transport structures in the attacked pith may have blocked the translocation of nicotine from roots to the shoots because pith in the RSJ is normally completely consumed by *T*. *mucorea* 2 weeks after infestation, destroying the intercalated phloem and xylem vascular bundles dispersed in the pith of many solanaceous plants [58]. Moreover, as reported from *N*. *sylvestris*, when nicotine transport from roots to shoots is blocked by blocking transpiration, de novo nicotine production is also truncated [59], indicating a feedback between nicotine biosynthesis and nicotine export from the root. Thus, whether the transport of nicotine in SL-RNAi plants is also attenuated and contributes to lower levels of inducible nicotine biosynthesis in roots needs further investigation.

JA signaling is well-known to promote nicotine production in *N*. *attenuata* [9,60]. In previous studies by Shoji and colleagues with cultivated tobacco, nicotine production is regulated by the JA/COI1/JAZ pathway, and MYC2 directly regulates JA-induced nicotine biosynthetic genes [61–63]. Hence, we expected that the higher JA levels in roots and pith of *max2*, *d14*, and *ccd7* plants should have increased nicotine contents of these tissues; this was clearly not the case. The unexpected combination of enhanced JA levels and reduced herbivore resistance of *max2*, *d14*, and *ccd7* plants was reminiscent of tomato SL-deficient plants, which have higher JA levels but are also more susceptible to RKN infections. Xu and colleagues proposed that this counterintuitive increase in susceptibility was due to the suppression of ABA by SL signaling. Because ABA is a negative regulator of defense against RKN infection, enhanced ABA levels in SL-deficient plants could contribute to the increased susceptibility by decreasing the accumulations of other defense compounds [39]. Here, nicotine was recovered in a JIC (JIC#1) (Fig 4D) in the metabolic network analysis; hence, we inferred that other factors were negating the positive effect of JA signaling. Indeed, three other phytohormones are known to regulate nicotine accumulations in various *Nicotiana* species: ABA, ethylene, and auxin [3,52,53,64,65]. By mining transcriptome data, we ruled out ABA- and ethylene-relevant genes (8 DEGs among 86 ABA-relevant annotated genes, 10 DEGs among 73 ET-relevant annotated genes) and found a strong auxin signal (17 DEGs among 65 IAA-annotated genes).

Auxin is well-known as a negative regulator of nicotine production in *Nicotiana* species [52,53,66]. In tobacco, IAA applications to wounds inhibit nicotine accumulation in leaves [53]. Moreover, removal of the shoot apex, a commonly used procedure in the cultivation of tobacco, increases nicotine concentrations in leaves, stem, and roots [52,66]. The nicotine levels induced by removal of the shoot apex are much larger than those commonly induced by leaf wounding, as we showed in an early study with a native population of *N*. *attenuata* [67]; this suggests that nicotine production is highly dependent on the major sources of auxin in the shoot apex [52]. After exogenous application of auxin, the nicotine biosynthetic genes *A622* and *PMT* are down-regulated in tobacco roots [66]. In this study, we showed that IAA levels decreased and nicotine levels increased in EV plants after CFM treatment for 3 days, without any changes in the number of branches (Fig 8H). In a preference assay with CFM-treated stems, more than 70% of the *T*. *mucorea* larvae preferred to feed on the pith of plants subjected to a CFM treatment than from the pith of control plants. Furthermore, the transcript levels of *A622* and *BBL* in EV plants were significantly reduced after 8 hours of IAA treatment (Fig 8I). From these results, we infer that the cross talk of SL and auxin clearly regulates nicotine accumulations on a short time scale, one that is independent of the developmental changes (branching of roots and shoots) observed in the SL-RNAi plants. We further demonstrated that the effect of auxin on nicotine biosynthesis is at least partially JA independent, as in the JA-deficient *aoc* plants, nicotine accumulations are still induced after decapitation, and in *aoc* seedlings, decreases in *A622* and *BBL* transcription abundances in response to IAA treatments are clearly seen (Fig 8I and 8J). However, the precise mechanism by which elevated auxin increases nicotine production independently of JA remains to be explored. In the pith of SL-RNAi plants, the inhibitory effect of high auxin levels clearly overrides the positive effects of JAs on nicotine accumulation.

The cross talk of JAs and auxin is also seen in the anthocyanin data: anthocyanin levels in EV×*d14* and EV×*max2* stems were significantly higher than in *aoc*×*d14* and *aoc*×*max2* plants (Fig 6A and 6B), suggesting a dominant effect of JAs on anthocyanin levels. Because IAA levels were higher in the stems of *aoc*×*d14* than in *aoc* plants (Fig 9A), the remaining high anthocyanin production in *aoc*×*d14* stems implied an effect of auxin in triggering anthocyanin accumulation, which is in line with the requirement of an auxin burst for the induction of anthocyanin in the stems of *N*. *attenuata* after *M*. *sexta* attack [5]. From these results, we conclude that the enhanced anthocyanin production in the stems of SL-RNAi plants results from elevated JA levels as well as IAA levels (Fig 9B). Statistical tertiary relationship analyses parsed the importance of SL as a regulator in fine-tuning defensive metabolites by disrupting the homeostasis of JA-IAA signaling (Fig 9).

This work reveals some of the molecular mechanism responsible for the apparent SL-JA cross talk. The up-regulated levels of SMXL6/7 in SL-RNAi plants (but not *kai2*) interact with specific JAZs to accelerate the degradation of JAZs. This releases the suppression of MYC2 by the JAZs to subsequently trigger JA signaling (Fig 7). How increased SMXL levels promote JAZ degradation and how SMXL6/7 interfere with the MYC2-JAZ interaction are some of the many remaining open questions. The amplified JA signaling in SL-RNAi plants in turn activates downstream genes, including *MYB8*, which amplifies specific sectors of phenylpropanoid metabolism to increase accumulations of phenolamides and anthocyanins (Figs 2 and 5). Genetic hybridizations revealed that JA signaling functions downstream of SLs to regulate anthocyanin and phenolamide accumulations (Fig 6). Phenolamides are known to function defensively against chewing folivores [47], and *myb8* plants, which are attenuated in their phenolamide accumulations, are more susceptible to foliar herbivores, such as the larvae of *M*. *sexta* and *Spodoptera littoralis* moths [47]. Interestingly, the stem-boring weevil, *T*. *mucorea*, performed similarly when infesting stems of *myb8* and EV plants (Fig 5C) [11], implying that phenolamides do not significantly contribute to the defense of *N*. *attenuata* stems against attack from *T*. *mucorea* larvae. The ecological consequences of increased phenolamides in SL-insensitive and -deficient plants need further study. Phenolamide levels in the leaves of SL-RNAi plants are possibly also increased by the amplified JA response, which might augment the resistance of these plants against attack from foliar herbivores.

Apart from the metabolites that we analyzed here (nicotine, phenolamides, and anthocyanin), other antinutritive metabolites that influence *T*. *mucorea* performance are also likely altered in SL-RNAi plants. Lignin, which controls the mechanical strength of cell walls and acts as a general antiherbivore defense [68], for instance, negatively affects the choice of *T*. *mucorea* weevils in pith-preference assays [41] and may well be differentially regulated in SL-deficient and -insensitive plants. Moreover, untargeted metabolic profiling conducted here revealed many mass features altered in RSJs of *max2* plants, and these features might reflect uncharacterized defenses against these insect larvae (Fig 4, S6 Fig). For instance, levels of mass features belonging to the JDC#2 module were strongly negatively correlated with JA levels (S6C Fig): in the RSJs of *max2* plants with high JA concentrations, levels of these features were much lower than in EV plants, whereas in the RSJs of JA-deficient *aoc* plants, these mass features were particularly abundant. These features might indicate a group of compounds that negatively regulate plant defense and affect the defense responses of *max2* plants.

The vast majority of research into plant–herbivore interactions is conducted with organisms that are convenient to study in the laboratory and are readily available as “reagent-grade” organisms available from stock centers and supply houses, but they represent only a small fraction of the different guilds of herbivores that interact with plants; namely, free-living folivores [69]. *T*. *mucorea* is an endophytic pith specialist that spends its entire 4-month larval and premating adult life sequestered inside the stem of annual *N*. *attenuata* plants. The larval development time alone takes a full month. Unlike free-living folivores, whose feeding triggers rapid, decisive responses in leaves, the endophytic lifestyle of *T*. *mucorea* means that the timing of the responses can be considerably slower. For endophytic interactions, in which the partners are not able to disengage from each other, the response times can last the lifetimes of the interacting partners. SLs, although not directly and rapidly activating the defenses that matter for weevil performance, use the existing JA and IAA regulatory networks to change the abundance of the relevant metabolites; this is an evolutionarily expected outcome, as evolution works by recombining and tinkering with existing elements [70]. Most whole-organismic interactions evolve from a network of interactions that might be considered “indirect” by engineers but rather have very direct fitness consequences for the organism and hence are subject to natural selection. The demonstration that the JA-regulated foliar defense metabolites, phenylamides, proven defenses against folivores in this system [47], are of no consequence to the weevils, highlights the importance of understanding these “indirect” roles of SL signaling and not just their “direct” interactions. This study shows that SL signaling tunes specific sectors of secondary metabolism by tailoring cross talk with JA and auxin; these SL effects may well be indirect consequences of the SL deficiencies in the SL-RNAi plants, indirect effects that alter auxin levels.

SL’s role in a plant’s responses to herbivore attack may not be limited to its regulation of metabolites that mediate resistance; it may also involve architectural responses associated with a plant’s tolerance of herbivore attack. A similar association has been reported in the gain-of-function mutant in rice, *ideal plant architecture 1* (*ipa1*), which decreases tillering but also increases resistance to a pathogen [71]. Plant architecture is known to play an essential role in a plant’s tolerance of herbivore attack [72–75]. Optimal defense theory predicts that defense and tolerance will be inversely related [76,77], but the molecular basis of such putative trade-offs remain unexplored. Increased branching is thought to provide greater tolerance of herbivore attack, as the additional meristems afforded by the increased branching allow plants to compensate for reproductive tissues lost to herbivores [72–74]. The maize ancestor, teosinte, with its greater number of branches—which are thought to result from a natural mutation in *TEOSINTE BRANCHED 1* (*TB1*), a transcription factor downstream in SL signaling [78]—exhibits a higher tolerance of attack from the stem-borer *Diatraea grandiosella* (Lepidoptera: Crambidae) with lower grain-production reductions relative to modern maize [79]. It is also interesting to note that this stem-boring moth and another *Sphenophorus* spp. weevil (Coleoptera: Curculionidae) were most frequently found on teosintes, whereas free-living aphids, thrips, leafhoppers, and fall armyworms were most abundant on maize [80], suggesting that different herbivore guilds have been recruited to the different growth forms. In *N*. *attenuata*, the levels of SLs are markedly reduced after *T*. *mucorea* attack (Fig 3H), suggesting a physiological preparation for a switch to a bushier architecture. Although we did not observe an increase in primary branch number after attack, this might simply reflect the short duration of the experiments conducted here, in which branch numbers were quantified for only 3 weeks after inoculation, and that experiments were conducted in the glasshouse with pot-grown plants, which are known to have attenuated branching compared with field-grown plants. Additional field experiments will be needed to more fully characterize the responses of SL-insensitive plants to herbivore attack. In conclusion, we propose that SLs may be mediating a switch between defense and tolerance to herbivore attack, and this exciting prospect, if correct, could provide a novel strategy for crop breeding.

## Materials and methods

### Plant material and growing conditions

In all experiments, WT *N*. *attenuata* Torr. Ex Watts seeds of the 31st generation inbred line were used. Seed germination and plant growth were performed as described [81]. *NaMAX2/D14/KAI2*-silenced lines were produced by the published *Agrobacterium tumefaciens*–mediated transformation method [81] using pRESC8 binary vector containing the inverted repeat (ir) fragment of the *NaMAX2* (NIATv7_g10118, NIATv7_g01142) sequences, *NaD14* (NIATv7_g26688, NIATv7_g08776) sequences, or *KAI2* (NIATv7_g33099, NIATv7_g21279) sequences. *CCD7*-silenced lines were constructed by using pSOL8 vector with *NaCCD7* (NIATv7_g01914) sequences. T_2_ plants were used in all experiments. The number of insertion copies as well as the fidelity of the insertion (over-reads and truncations) were evaluated by NanoString [42]. In brief, DNA extracted from individual transgenic plants was used for NanoString to detect the content of inserted fragments according to the designed probes. To validate the single-copy-number insertions inferred from the segregation rates, a published single-copy insertion stably transformed line, *ago8* (871-8-8), was used as a positive control [82]. The following three previously characterized transgenic lines were also used in this study: *myb8* (A-07-810-2) plants silenced in the expression of *MYB8* gene [47]; *pmt* (A-03-108-3) plants silenced in the expression of putrescine N-methyltransferase, a key nicotine biosynthetic enzyme [51]; and *aoc* (A-07-457-1) plants silenced in the expression of AOC, a single-copy key biosynthetic enzyme in JA biosynthesis [45].

All experiments were performed in the glasshouse. *N*. *attenuata* plants were grown with a day/night cycle of 16 hours (26–28°C)/8 hours (22–24°C). For decapitation experiments and CFM treatments, plants with eight leaf nodes on the stem were used. The top two nodes with unexpanded small leaves were removed in the decapitation experiment, and eggs of *T*. *mucorea* were inoculated on the same day. Plants treated with CFM (1 μg/mL) on the second node (from bottom to above) for 3 days were used in pith-preference assays.

### Anthocyanin measurement

The epidermis of stems of 75-day-old plants (Fig 2) and 90-day-old plants (Fig 6) was removed, and anthocyanin levels were measured by Dualex (FORCE-A).

### *T*. *mucorea* performance and pith-preference assays

*T*. *mucorea* eggs were inoculated into *N*. *attenuata* plants as described [11]. In brief, freshly collected eggs were inoculated into the petiole of a healthy rosette leaf located at the basal stem (RSJ) of a plant in the early elongation stage of growth. After the indicated inoculation period (approximately 1–3 weeks), egg-inoculated stems were split to harvest plant material or collect larvae for weighing or pith-preference assays, as previously described [41]. Approximately 5 cm of pith section above the area attacked by *T*. *mucorea* larvae and control samples consisting of a 5-cm pith section just above the RSJ were harvested for analysis and immediately frozen in the liquid nitrogen.

For pith-preference bioassays, the stems of the different genotypes were split lengthwise and paired with size-matched halves of stems from EV plants in the same stage of growth. Larvae harvested from 2-week-inoculated *max2* plants were individually placed into an approximately 1-mm^3^ hole excavated in the pith of the RSJ on both halves of the stem “sandwich.” The two halves were wrapped with M3 tape to hold the two halves together. Stems were kept hydrated and held in a vertical position by covering them with wet tissues. After 36 hours, the tape wrapping was removed, and both stem halves were inspected for feeding damage. The volume of the consumed pith area was scored as a criterion for the feeding choice of larvae.

### Total RNA extraction and transcript abundance analysis

For transcript abundance analysis, RNA was extracted from the indicated tissues using the RNAeasy Plant Mini Kit (Qiagen) or NucleoSpin RNA Plant (Macherey-Nagel) according to the manufacturer’s instructions. Isolated RNA was used for microarray or reverse transcription. cDNA was prepared by using the PrimeScript RT-qPCR Kit (TaKaRa). For microarray analysis, extracted RNA was labeled and hybridized according to the protocol of the Quick Amp labeling kit (Agilent, http://www.agilent.com/home). Agilent single-color technology arrays (60 k) were used for the hybridizations. Raw intensity data were normalized with the quantile method, and subsequently, the low expression probes were discarded after log2 transformation. Differentially expressed probes were filtered after pairwise comparisons (|log2FC| ≥ 1, FDR ≤ 0.05).

RT-qPCR was performed on a Stratagene Mx3005P qPCR machine using a SYBR Green containing reaction mix (Eurogentec, qPCR Core kit for SYBR Green I No ROX). For all qPCR analysis, *N*. *attenuata IF-5A* housekeeping gene was used as internal reference. Primer sequences were listed in the S2 Table.

### Primary and secondary metabolites extractions and analysis

For primary and secondary metabolites extraction, ground plant material was aliquoted into Eppendorf tubes, and their masses were recorded for normalization. Per 100 mg of plant tissues, 800 μL of extraction buffer (0.2 N formic acid in 80% MeOH with internal standards) was pipetted into the samples before being shaken in a GenoGrinder 2000 (SPEX SamplePrep) for 60 seconds at 1,150 strokes. After two centrifugations, the supernatant was collected, and 100 μL was aliquoted for secondary metabolites analysis. The remaining supernatant was used for further hormonal extraction and analyzed following the procedures described by Schäfer and colleagues [83].

For root exudate collections and SL measurements, 20-day-old *N*. *attenuata* plants were transferred individually to 1-L pots containing hydroponic solution. After 20 days, the hydroponic solutions were replaced by solutions depleted in inorganic Pi for a week for *max2* and *d14* plants and 10 days for *ccd7* plants. Then 200 mL of hydroponic solution was collected and acidified by adding 200 μL of formic acid. The collected volume of hydroponic solution was passed through an HR-X column (Macherey-Nagel). The column was subsequently washed with 2 mL of water and finally eluted with 2 mL of 100% MeOH. Before chromatographic measurement, the dehydrated precipitate was resuspended with 200 μL of 80% MeOH.

For chromatographic separations, UHPLC (Dionex UltiMate 3000) equipped with a reverse phase column (Agilent ZORBAX Eclipse XDB C18, 50 × 3.0 mm, 1.8 μm) as the stationary phase was used. Mass spectrum (MS) detection for primary metabolites analysis was performed on a Bruker Elite EvoQ triple-quadrupole MS equipped with a heated electrospray ionization (HESI) ion source; MS detection for secondary metabolites analysis was performed on a microTOF-Q II MS system (Bruker Daltonics) equipped with an electrospray ionization (ESI) source operating in positive ion mode. Additionally, for nicotine quantification, samples were measured by microTOF-Q II MS described previously [46]. For 2′-*epi*-orobanchol quantification, purchased standards including strigol, orobanchol, and 2′-*epi*-orobanchol from OlChemIn (www.olchemin.cz) together with concentrated root exudates were analyzed by microTOF-Q II MS, and the retention times of extracted ion chromatograms (*m/z* 347.15 ± 0.05) were compared. For the fragmentation of the proposed daughter ions via pseudo-MS^3^, we applied a 20-eV in-source-CID transfer energy, which produced spectra reflecting the loss of the butenolide moieties.

### Metabolome analysis

Raw data collected from the LC-MS instrument were preprocessed with R packages “XCMS” and “CAMERA” without fillPeak function. A missing value (MV) filtering rule was applied across individual samples or single peaks (cutoff: 0.5, 0.5), and 5,649 mass features were retained. An MV imputation with a minimum value across samples for each feature was implemented, after which a 75% percentile normalization and log2 transformation scaling was followed. “NA” were MVs, which were filtered by applying the following rules: if the proportion of MV was greater than 50% in each row (features) or column (samples), such features or samples were removed from the analysis, and 5,649 mass features were retained. Remaining “NA" MVs were replaced with the minimum value of each row. A 75% percentile normalization process was conducted, and log2 transformation was followed for the WGCNA pipeline. PLS-DA methods were applied for PCA analysis to classify batch and genotype effects. Prior to WGCNA processing, statistical filtering was performed according to comparisons of any group relative to EV as a reference group in the same batch. Significant differences were defined as (|log2FC| > 0.58, FDR < 0.05). Eventually, 535 features were used for the WGCNA pipeline (S1 Table), which used the following parameters: signed as TOMType = “unsigned”, power = 10, minModuleSize = 30, and corType = “bicor” and then output TOM > 0.3 nodes and edges as Cytoscape network visualization input. Correlations were computed between pith JA contents and extracted subnetworks, respectively; means of Pearson’s correlation coefficients and *P* values are reported. For ternary analysis, medians of features from each genotype were extracted and scaled as input variables, and the R packages “VCD” and “ggtern” were used for plotting.

### Phylogeny analysis

Protein sequences of indicated genes were downloaded from NCBI or SGN (https://solgenomics.net) and *N*. *attenuata* Data Hub (http://nadh.ice.mpg.de/NaDH/others/genes). MEGA5 was used for phylogenic analysis; in brief, after alignment by using MUSCLE algorithm, a maximum likelihood tree was generated with default parameters (JTT algorithms).

### Cloning

For transient expression experiments, *SMXL6* and *SMXL7* were cloned into pBA-YFP; *MAX2a* and *MAX2b* were cloned into p1302SNB to generate C-terminal YFP and eGFP fused genes. *JAZb* was cloned into pEarleyGate 203 to generate N-terminal myc fused gene. Constructs were transformed into *A*. *tumefaciens* GV3101. For Y2H assay, *SMXL6*, *SMXL7*, and *D14* were cloned into pGBKT7 to generate BD fused constructs; each *JAZ* was cloned into pGADT7 to generate AD fused genes. *MAX2a* and *MAX2b* genes were cloned into pGADT7, respectively, to generate AD fused genes via NdeI/SmalI digest; A*SK1* (NIATv7_g32726) was then cloned into *AD*-*MAX2a* and *AD*-*MAX2b* vectors to generate *AD-ASK1-MAX2a* and *AD-NSK1-MAX2b* vectors via NdeI digest. For prokaryotic expression, *JAZb*, *JAZk*, and *D14* were cloned into pDEST–N112–MBP and pDEST15 to generate HIS–MBP and GST fused gene, respectively. Constructs were transformed into *Escherichia coli* BL21 (DE3).

### Subcellular localization

*Agrobacterium* containing 35S: SMXL6-YFP, 35S: SMXL7-YFP, 35S: D14-YFP, 35S: MAX2a-eGFP, 35S: MAX2b-eGFP, or nuclei-localized marker 35S: SV40-CFP construct was infiltrated into *N*. *benthamiana* leaves. Three days after incubation, fluorescence was analyzed by confocal microscopy.

### Y2H and Y3H assays

Y2H assay was performed by using Matchmaker Gold Yeast Two-Hybrid System (Clontech) under the manufacturer’s instruction. In brief, AD and BD fusions constructed along with their own EVs as control were cotransformed into freshly made Y2H gold competent cells and plated on selective dropout medium (SD-Leu/-Trp). The transformations grew on QDO (SD-Leu/-Ade/-His/-Trp) medium in the presence of X-α-gal (40 μg/mL) and 2 mM 3AT or *rac*-GR24 (5 μM) if needed at 30°C for 5–7 days after incubation for recording.

In Y3H assays, pBridge-JAZb-Promoter (met)-SMXL6/7 and pGADT7-MYC2a/b were used. The expression of SMXL6/7 was induced by adding decreasing amounts of Met as indicated. Transformed yeast were grown on selective dropout medium (SD-Leu/-Trp) with AbA (70 μg/mL). The transformations grew on QDO (SD-Leu/-His/-Trp/-Met) medium in the presence of X-α-gal (40 μg/mL) and AbA (70 μg/mL) with the addition of different amounts of Met. After incubating at 30°C for 5 days, the growth of yeast was recorded.

### Purification of recombinant protein

Single clone of His-MBP-JAZb, His-MBP-JAZk, and D14-GST was respectively cultured in 1 mL Luria–Bertani (LB) at 37°C for 24 hours, and then 10 μL of the culture was transferred to 500 mL LB medium for amplification, and then 0.5 mM isopropyl-β-thiogalactopyranoside (IPTG) was added when OD_600_ reached around 0.4 for an additional 3 hours at 37°C. HIS-tagged protein was purified using Ni-NTA agarose (Qiagen), and GST-tagged protein was purified using Glutathione Sepharose 4B (GE Healthcare) according to the respective manufacturer’s manual.

### Pull-down assays

*N*. *benthamiana* leaves were infiltrated with *Agrobacterium* containing either 35S: SMXL6-YFP or 35S: SMXL7-YFP. Leaves were harvested after 72 hours of incubation in climate chambers at 20–22°C. Approximately 3 g of leaf material was extracted in 2.4 mL of extraction buffer (50 mM Tris-Cl [pH 6.7], 100 mM NaCl, 10% [v/v] glycerol, 0.1% [v/v] Tween 20, 20 mM 2-mercapto-ethanol, and 10 μM MG132), and 0.8-mL extracts were added into 25-μL GFP-Trap-A beads (Chromotek) and incubated at 4°C for 1 hour. Supernatant was then removed after short spin, and these steps were repeated several times. A three-times washing step was followed by using pull-down binding buffer (50 mM Tris-Cl [pH 7.0], 150 mM NaCl, 0.5% Tween 20), and then 1 μg of purified D14-GST was added into the reaction system containing protease inhibitor mixture and 10 μM MG132 with/without 20 μM *rac*-GR24, and incubation was performed at 22°C for an additional hour. After washing five times by using pull-down washing buffer (50 mM Tris-Cl [pH 7.0], 300 mM NaCl, 0.5% Tween 20) and carefully removing supernatant, 100 μL 2× SDS loading buffer was pipetted into it and boiled at 95°C for 5 minutes. Then, 20 μL was used by SDS-PAGE and immunoblotted using anti-GST antibody (Sigma).

### Co-IP assay

*N*. *benthamiana* leaves were coinfiltrated with *Agrobacterium* containing genes encoding 35S: MYC-JAZb along with either 35S: SMXL6-YFP or 35S: SMXL7-YFP. Leaves were harvested after 72 hours of incubation at 20–22°C. Approximately 5 g of leaf material was extracted in 4 mL of extraction buffer, and 50-μL GFP-Trap-A beads were added into 1-mL extracts; the incubation and sample preparation process was the same as described in the pull-down assay and immunoblotted using anti-myc antibody (Sigma).

### *In vitro JAZ* degradation assay

Pith material just above RSJ was harvested from adult plants at flowering stage of EV and *max2* plants; around 200 mg of plant materials was used for total protein extraction. Crude protein was adjusted to the same concentration (0.5 μg/μL). Recombinant His-MBP-JAZb (5 μg) was added into crude protein from indicated plants and incubated at 22°C for the indicated times. Samples were harvested at each time point for western blot using anti-His antibody.

### Accession numbers

Sequence data from this article can be found in the *N*. *attenuata* Data Hub under the following accession numbers: *JAZa* (NIATv7_g42021), *JAZb* (NIATv7_g23423), *JAZc* (NIATv7_g08281), *JAZd* (NIATv7_g10090), *JAZe* (NIATv7_g17159), *JAZf* (NIATv7_g25875), *JAZg* (NIATv7_g36100), *JAZh* (NIATv7_g26197), *JAZi* (NIATv7_g29371), *JAZj* (NIATv7_g27995), *JAZk* (NIATv7_g32564), *JAZl* (NIATv7_g38280), *JAZm* (NIATv7_g34582), *SMXL6* (NIATv7_g27847), *SMXL7* (NIATv7_g37691), *MAX2a* (NIATv7_g10118), *MAX2b* (NIATv7_g01142), *D14*(NIATv7_g26688), *CCD7* (NIATv7_g01914), *CCD8* (NIATv7_g12116), *BBL* (NIATv7_g17187), *A622* (NIATv7_g09977), *MYB8* (NIATv7_g41919) and *DH29* (NIATv7_g06682).

## Supporting information

S1 Fig Characterization of *MAX2* gene in *N. attenuata*. (A) Phylogenetic analysis of *MAX2* gene family from CA, Na, Ph, Sl, and St. (B) Subcellular localization of *MAX2a* and *MAX2b*. MAX2a-eGFP and MAX2b-eGFP were transiently expressed in *N*. *benthamiana* leaves 72 hours after inoculation. Scale bar, 10 μm. (C) Interactions between NaMAX2a/b and NaD14 proteins in the presence of *rac*-GR24 as evaluated by yeast two-hybrid assays. GAL4 DNA-BD-NaD14 and AD-NaMAX2a/2b were cotransformed into yeast. The transformants were grown on QDO (SD −Ade/−His/−Leu/−Trp). (D) Vector map of pRESC8MAX2 transformation construct. Primers designed within the LB and RB region (blue) and outside the LB and RB region (red) were used in (F). (E) Relative signal intensity of *max2*#1 and *max2*#2 plants with indicated primers. A published single-copy insertion of the stably transformed line (*ago8*) was used as a positive control. The single and complete T-DNA insertion in both *max2*#1 and *max2*#2 plants was validated by using NanoStrings nCounter technology as described in [42]. Briefly, TNOS+LB_1, hptII_3, PNOS_1, P35S_1, and T35S_1 (blue) are used to evaluate single and complete T-DNA insertions. pVS1_3 and nptII_1 (red) are probes indicating over-reads of the plasmid sequences outside of the T-DNA borders, which do not affect the plant phenotype or its stable inheritance; note that the lack of signal from these probes reveals that over-reads did not occur during the transformation process. (F) Representative hypocotyl phenotypes of EV, *max2*#1, and *max2*#2 seedlings after growth with 5 μM *rac*-GR24 or 5 μM KAR1 treatment for 7 days. Scale bar, 5 mm. Hypocotyl length of EV, *max2*#1, and *max2*#2 seedlings without treatment and relative hypocotyl length of EV, *max2*#1, and *max2*#2 seedlings with indicated treatment (±SE, *n =* 11–15). (G) Seed germination ratios of EV, *max2*#1, and *max2*#2 seeds with KAR1 (0.01 ng/μL) treatment for 9 days (±SE, 4 replicates, each replicates included 25 seeds) (*P < 0.05; **P < 0.01; ***P < 0.001; two-tailed Student *t* test). Values for graphs (D-G) are listed in S1 Data. AD, activation domain; *ago 8*, *argonaute 8*; BD, binding domain; CA, *Capsicum annuum*; D14, dwarf14; eGFP, enhanced green fluorescent protein; EV, empty vector; KAR, karrikin; LB, left border; MAX2, more axillary growth 2; Na, *N*. *attenuata*; n.s., not significant; Ph, *P*. *hybrida*; RB, right border; Sl, *S*. *lycopersicum*; St, *S*. *tuberosum*; T-DNA, transfer DNA.
(TIF)

S2 Fig Characterization of *D14*, *CCD7*, and *KAI2* genes in *N. attenuata*. (A and B) Phylogenetic analysis of *D14* and *KAI2* (A), *CCD7* and *CCD8* (B) gene families from At, Os, Sl, Na, Ph, Ps, Mt, and Pt. (C and D) pRESC8D14 and pRESC8KAI2 were used for the construction of the *d14* and *kai2* lines. The basic architecture of the vector was the same as shown for the vector described in S1D Fig with *d14* and *kai2* fragments replacing the concatenated *max2* fragments. Relative signal intensity of *d14*#1 and *d14*#2 (C) and *kai2*#1 and *kai2*#2 (D) plants was quantified with the indicated primers. (E) Vector map of pSOL8CCD7 transformation construct used for constructing *ccd7* lines and relative signal intensity of *ccd7*#1 and *ccd7*#2 plants with indicated primers. Primers for constructing vectors are described in S2 Table. Values for graphs (C-E) are listed in S1 Data. At, *A. thaliana*; CCD, carotenoid cleavage dioxygenase; d14, dwarf14; max2, more axillary growth 2; Mt, *Medicago truncatula*; Na, *N*. *attenuata*; Os, *O*. *sativa*; Ph, *P*. *hybrida*; Ps, *Pisum sativum*; Pt, *Populus trichocarpa*; Sl, *S*. *lycopersicum*.
(TIF)

S3 Fig Orobanchol levels in root exudates of *max2*, *d14*, and *ccd7* plants. (A) A scheme of SL biosynthesis and perception in *N*. *attenuata*. (B) Relative transcript abundance of *CCD7* and *CCD8* in roots of EV, *d14*, and *max2* plants (±SE, *n =* 3–4). (C) Representative chromatograms of root exudates, (±)-2′-*epi*-orobanchol, (±) orobanchol, and (±) strigol analyzed under positive ionization mode. EIC: 347.15 ± 0.05. (D) Comparison of fragmentation patterns of root exudates and (±)-2′-*epi*-orobanchol by high-resolution tandem MS. (E) Representative chromatograms of (±)-2′-*epi*-orobanchol in root exudates, as analyzed by targeted UHPLC-triple quadrupole-MS metabolomics; different MRM ions were listed on the top of the panel. (F) (±)-2′-*epi*-orobanchol levels (Kcps) in root exudates of hydroponic EV, *max2*, *d14*, and *ccd7* plants (±SE, *n =* 3–4) were analyzed by targeted UHPLC-triple quadrupole-MS metabolomics. Quantifier ion (m/z) 347.1 > 233.1 was selected to achieve maximum signal intensity. Three independent experiments were performed at different developmental stages (two-tailed Student *t* test). Values for graphs (B) and (F) are listed in S1 Data. ccd, carotenoid cleavage dioxygenase; d14, dwarf 14; EIC, extracted ion chromatogram; EV, empty vector; Kcps, kilo counts per second; max2, more axillary growth 2; MRM, multiple reaction monitoring; SL, strigolactone; UHPLC, ultra-high-performance liquid chromatography.
(TIF)

S4 Fig Shoot architecture is not affected by *T*. *mucorea* attack. (A) Representative phenotypes of EV and *max2*#2 pith after attack by *T*. *mucorea* larvae for 3 weeks. The attacked area in the pith is outlined with red lines. Scale bar, 5 mm. (B) Stem diameter, plant height, and branch number per plant of EV and *max2*#2 plants without or with *T*. *mucorea* larvae attack (±SE, *n =* 10–20). (C) Representative images and results of *T*. *mucorea* attacked pith from pith-preference bioassays. Scale bar, 5 mm. For the preference bioassays, larvae were placed between two stem halves of either EV and EV, EV and *max2*#1, or EV and *max2*#2 plants. The amount of pith damaged in both halves was quantified after 36 hours (±SE, 3 replicates, each replicate includes 8–10 plants) (**P < 0.01; ***P < 0.001; two-tailed Student *t* test). Values for graphs (B) and (C) are listed in S1 Data. EV, empty vector; max2, more axillary growth 2; n.s., not significant.
(TIF)

S5 Fig Transcriptome analysis of genes expressed in RSJ of *max2* plants. (A) MDS plot of the gene expression data in the RSJs of EV (red), *max2*#1 (green), and *max2*#2 (blue) plants. Microarray analysis was performed for gene expression. Each line included four replicate plants. (B) Venn diagrams showing overlap among up-regulated and down-regulated genes in the RSJs of *max2*#1 and *max2*#2 plants. (C) Heatmap presents the expression of up-regulated and down-regulated genes in the RSJs of EV, *max2*#1, and *max2*#2 plants. The color gradient represents the relative sequence abundance. (D) GO enrichment of 1,160 up-regulated and 435 down-regulated genes in the RSJs of both *max2*#1 and *max2*#2 plants. (E) Relative transcript abundance of JA-related genes *OPR2a*, *OPR2b*, *JAR4*, and auxin-related genes *PIN1*, *PIN7*, *YUC6*, *PIN6*, and *AUX1*. Expression levels were analyzed from microarray data (±SE, *n =* 4). (F) Relative transcript abundance of *JAR4*, *PIN1*, *PIN7*, *YUC6*, and *AUX1* in RSJ of indicated plants. Expression levels were analyzed by RT-qPCR (±SE, *n =* 4) (*P < 0.05; **P < 0.01; ***P < 0.001; two-tailed Student *t* test). Values for graphs (E) and (F) are listed in S1 Data. *AUX1*, *AUXIN RESISTANT 1*; EV, empty vector; GO, Gene Ontology; JA, jasmonate; *JAR4*, *JASMONIC ACID RESISTANT 4*; MAX2, more axillary growth 2; MDS, multidimensional scaling; OPR, OPDA reductase; PIN, PIN-FORMED; RSJ, root–shoot junction; *YUC6*, *YUCCA 6*.
(TIF)

S6 Fig Coexpression network analysis of nontargeted metabolic profiling. (A) The reciprocal interactions amongst features (TOM > 0.3) was exported as visualization input by Cytoscape. Based on exported features in network, the coexpression patterns of the different modules are illustrated by heatmaps; the class of annotated *O*-acyl sugar specialized metabolites was specifically extracted from the blue module. Node colors indicate computed modules by WGCNA. (B and C) Extracted subnetworks of JDC#1 and JDC#2 visualized by Cytoscape (left panels) and correlations with JA contents with each subnetwork were calculated as Pearson’s correlation coefficients and their *P* values. Bar charts display the representative compounds from each subnetwork across all samples from two batches (dark gray: first batch; purple: second batch). Values for graphs (B) and (C) are listed in S1 Table. JA, jasmonate; JDC, JA-dependent cluster; TOM, topological overlap matrix; WGCNA, weighted correlation network analysis.
(TIF)

S7 Fig Identification of NaSMXL6/7 and NaD14. (A) Extended phylogenetic analysis for Fig 7B of *SMXL* gene family from Na (red), At, and Os. (B) Subcellular localization of NaSMXL6 and NaSMXL7. NaSMXL6-YFP or NaSMXL7-YFP were coinoculated with SV40-CFP and transiently expressed in *N*. *benthamiana* leaves for 72 hours. Scale bar, 10 μm. (C) Subcellular localization of NaD14. NaD14-YFP and SV40-CFP were coinoculated and transiently expressed in *N*. *benthamiana* leaves for 72 hours. Scale bar, 10 μm. (D) Protein levels of NaSMXL6 in leaves of EV, *max2*#1, and *max2*#2 plants. (E) JAZk degradation in EV and *max2* (#1, #2) crude proteins. Purified His-JAZk was incubated in EV or *max2* crude proteins extracted from the pith for the indicated times. His-JAZk were detected by anti-His. The CBB staining presents protein loading levels. (F) Extended results for Fig 7K of the interference of SMXL6/7 in the interactions of JAZb and MYC2a/b as revealed by Y3H assays. The expression of SMXL6/7 was gradually induced by decreasing concentrations of Met. Raw images for blots are listed in S1 Raw Images. At, *A*. *thaliana*; CBB, Coomassie Brilliant Blue; D14, DWARF14; EV, empty vector; JA, jasmonate; JAZ, jasmonate zim-domain; MAX2, more axillary growth 2; Met, methionine; Na, *N*. *attenuata*; Os, *O*. *sativa*; SMXL, suppressor of max2-like; YFP, yellow fluorescent protein.
(TIF)

S8 Fig Nicotine responses to *T*. *mucorea* attack. (A) Relative content of nicotine in the pith of EV and *max2*#2 plants with or without *T*. *mucorea* attack for 2 weeks (±SE, *n =* 5–10). (B) Relative contents of nicotine in roots of EV, *max2*#1, and *max2*#2 plants with or without *T*. *mucorea* attack (±SE, *n =* 6–8). (C) Relative contents of JA and IAA in roots of EV, *max2*#1, and *max2*#2 plants (±SE, *n =* 6). (D) Relative transcript abundance of nicotine biosynthetic genes *BBL* and *A622* in EV and *max2*#2 seedlings with or without 10 μM IAA treatment for 8 hours. Transcripts levels were quantified by RT-qPCR (±SE, *n =* 4). (E) Nicotine contents in the RSJs of *max2*#2 plants with or without decapitation treatments for 3 days (*n =* 8) (two-tailed Student *t* test). Values for graphs (A–E) are listed in S1 Data. EV, empty vector; IAA, indole-3-acetic acid; JA, jasmonate; MAX2, more axillary growth 2; RSJ, root–shoot junction.
(TIF)

S1 Table Data for untargeted metabolic analysis. In total, 535 differentially expressed features with their abundances across all 41 samples for WGCNA application as presented in Fig 4 and S6 Fig. WGCNA, weighted correlation network analysis.
(XLSX)

S2 Table Primers used in this study. All sequence information of oligonucleotide primers used in this study.
(XLSX)

S1 Data Numerical raw data used to create the graphs in the paper. (XLSX)

S1 Raw Images Uncropped blots for western blot shown in the paper. (PDF)

## Abbreviations

ABA: abscisic acid
ACS2: 1-aminocyclopropane-1-carboxylic acid synthase
AMF: arbuscular mycorrhizal fungi
AOC: allene oxide cyclase
At: *Arabidopsis thaliana*
AUX1: auxin resistant 1
BES1: *bri1*-EMS-suppressor 1
BR: brassinosteroid
BRC1: branched 1, CCD, carotenoid cleavage dioxygenase
CFM: methyl-2-chloro-9-hydroxyfluorene-9-carboxylate
CGA: chlorogenic acid
CK: cytokinin
COI1: coronatine insensitive 1
Co-IP: coimmunoprecipitation
CP: *N*′-caffeoylputrescine
D14: dwarf 14
D27: dwarf 27
DCS: *N*′, *N″*-decaffeospermidine
DEG: differentially expressed gene
ein: ethylene insensitive
etr: ethylene receptor
EV: empty vector
FDR: false discovery rate
GA: gibberellin
GO: Gene Ontology
HGL-DTG: 17-hydroxygeranyllinalool diterpene glycoside
IAA: indole-3-acetic acid
ipa1: ideal plant architecture 1;JA, jasmonate
JA-Ile: jasmonic acid-isoleucine
JAR4: jasmonic acid resistant 4
JAZ: jasmonate zim-domain
JDC: JA-dependent cluster
JIC: JA-independent cluster
KAI2: karrikin insensitive 2
KAR: Karrikin
LBO: lateral branching oxidoreductase
LC: liquid chromatography
LOX: lipoxygenase
MAX2: more axillary growth 2
MDS: multidimensional scaling
Met: methionine
Na: *Nicotiana attenuata*
OPR: OPDA reductase
PIN: pin-formed
PLS-DA: partial least squares discriminant analysis
pmt: putrescine n-methyltransferase
qTOF-MS: quadrupole time-of-flight mass spectrometry
RNAi: RNA interference
RKN: root-knot nematode
RSJ: root–shoot junction
SA: salicylic acid
SCF: Skp1–Cullin–F-box
Skp1: s phase kinase-associated protein 1
Sl: *Solanum lycopersicum*
SL: strigolactone
SLR1: pseudogene of S-locus related protein
SMXL: suppressor of max2-like
TB1: teosinte branched 1
TPI: trypsin proteinase inhibitor
TOM: topological overlap matrix
VIGS: virus-induced gene silencing
WGCNA: weighted correlation network analysis
wpi: weeks postinoculation
WT: wild-type
Y2H: yeast two-hybrid
YUC6: yucca 6
