# Supplementary figures and images for: Strigolactone signaling regulates specialized metabolism in tobacco stems and interactions with stem-feeding herbivores
Source: PLoS Biol. 2020 Aug 18;18(8):e3000830. doi: 10.1371/journal.pbio.3000830 (PMC7478753; doi:10.1371/journal.pbio.3000830)

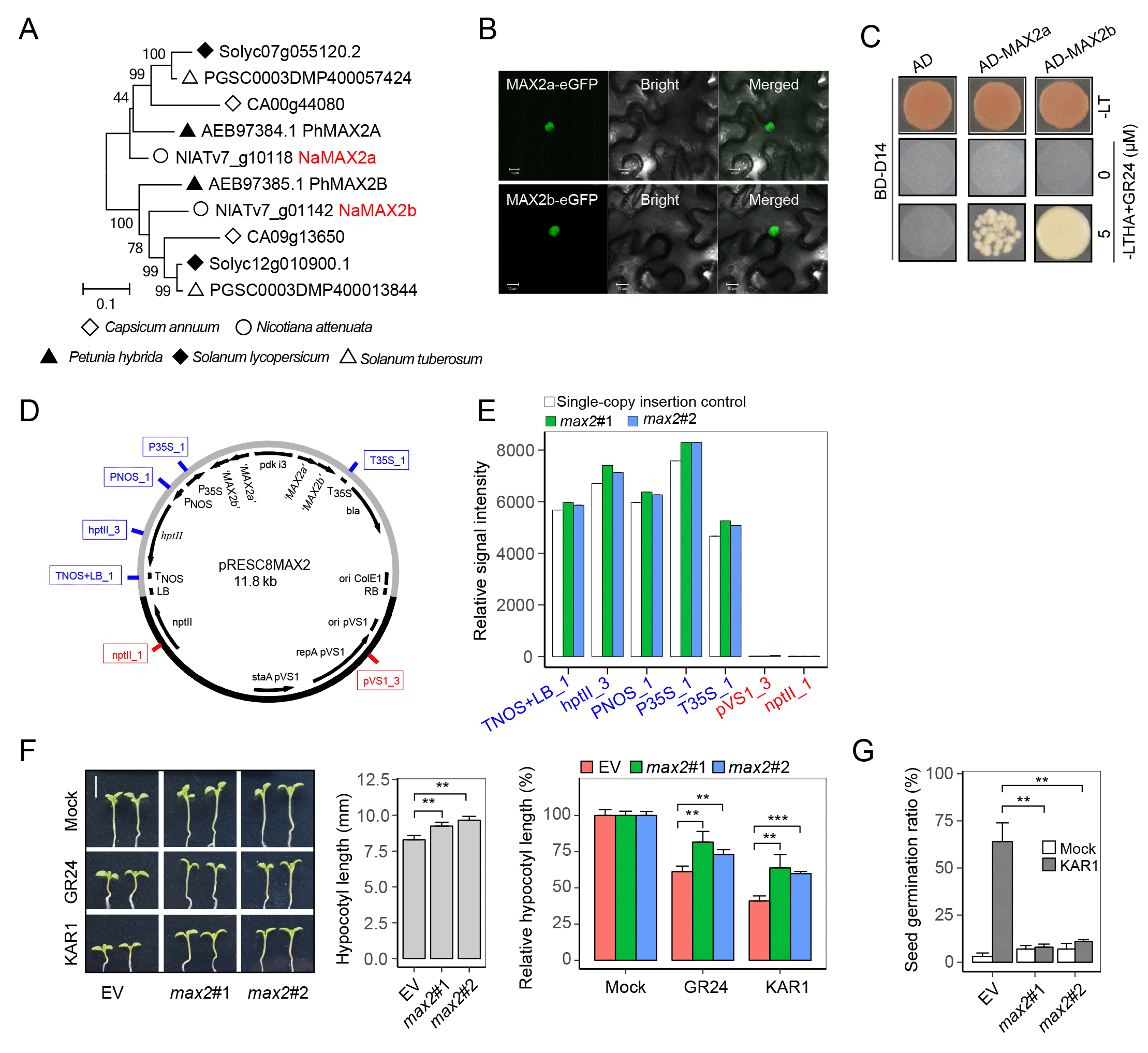

Supplement: S1 Fig — (A) Phylogenetic analysis of MAX2 gene family from CA, Na, Ph, Sl, and St. (B) Subcellular localization of MAX2a and MAX2b. MAX2a-eGFP and MAX2b-eGFP were transiently expressed in N. benthamiana leaves 72 hours after inoculation. Scale bar, 10 μm. (C) Interactions between NaMAX2a/b and NaD14 proteins in the presence of rac-GR24 as evaluated by yeast two-hybrid assays. GAL4 DNA-BD-NaD14 and AD-NaMAX2a/2b were cotransformed into yeast. The transformants were grown on QDO (SD −Ade/−His/−Leu/−Trp). (D) Vector map of pRESC8MAX2 transformation construct. Primers designed within the LB and RB region (blue) and outside the LB and RB region (red) were used in (F). (E) Relative signal intensity of max2#1 and max2#2 plants with indicated primers. A published single-copy insertion of the stably transformed line (ago8) was used as a positive control. The single and complete T-DNA insertion in both max2#1 and max2#2 plants was validated by using NanoStrings nCounter technology as described in [42]. Briefly, TNOS+LB_1, hptII_3, PNOS_1, P35S_1, and T35S_1 (blue) are used to evaluate single and complete T-DNA insertions. pVS1_3 and nptII_1 (red) are probes indicating over-reads of the plasmid sequences outside of the T-DNA borders, which do not affect the plant phenotype or its stable inheritance; note that the lack of signal from these probes reveals that over-reads did not occur during the transformation process. (F) Representative hypocotyl phenotypes of EV, max2#1, and max2#2 seedlings after growth with 5 μM rac-GR24 or 5 μM KAR1 treatment for 7 days. Scale bar, 5 mm. Hypocotyl length of EV, max2#1, and max2#2 seedlings without treatment and relative hypocotyl length of EV, max2#1, and max2#2 seedlings with indicated treatment (±SE, n = 11–15). (G) Seed germination ratios of EV, max2#1, and max2#2 seeds with KAR1 (0.01 ng/μL) treatment for 9 days (±SE, 4 replicates, each replicates included 25 seeds) (*P < 0.05; **P < 0.01; ***P < 0.001; two-tailed Student t test). Values for gr [file pbio.3000830.s001.tif]

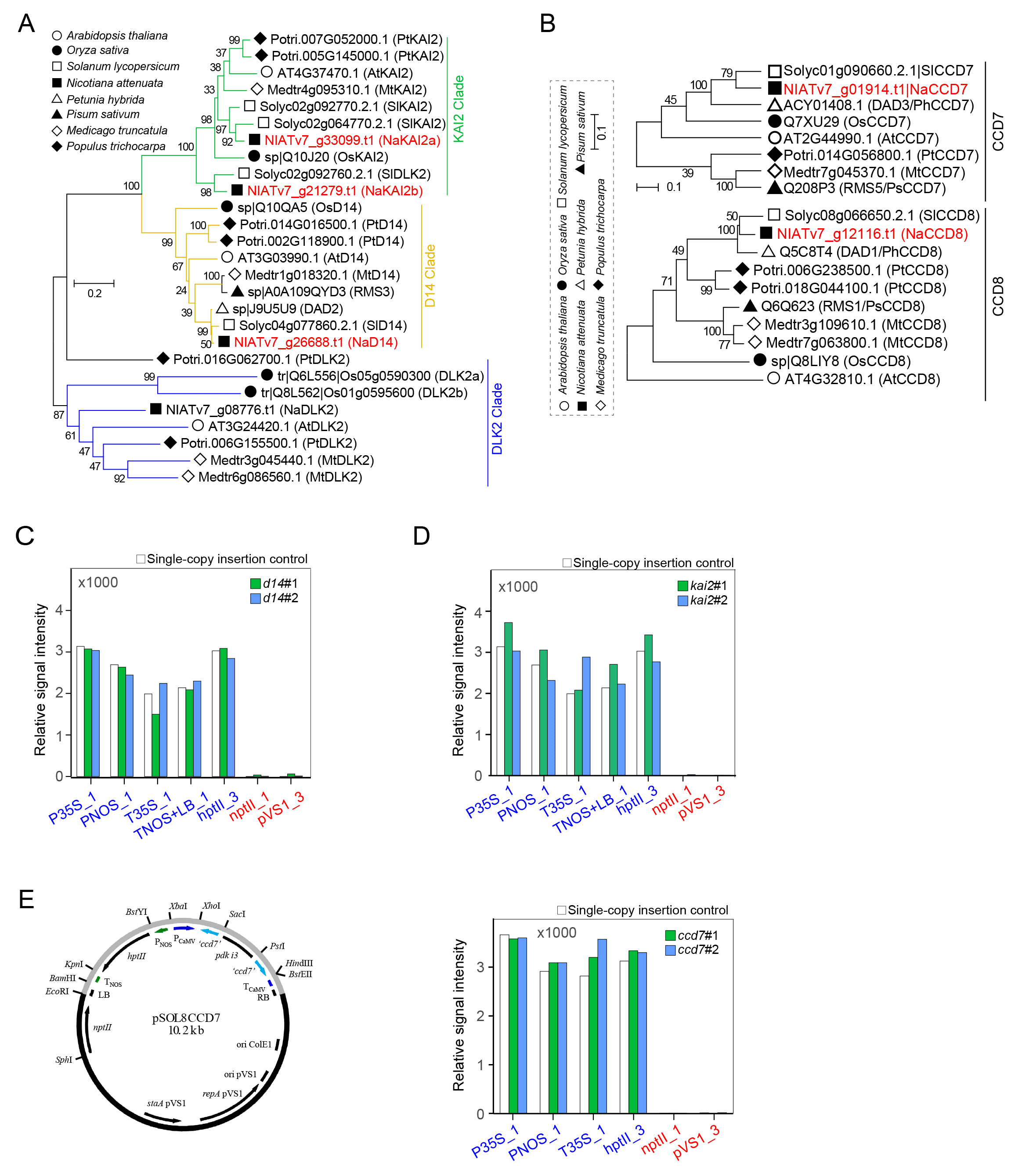

Supplement: S2 Fig — (A and B) Phylogenetic analysis of D14 and KAI2 (A), CCD7 and CCD8 (B) gene families from At, Os, Sl, Na, Ph, Ps, Mt, and Pt. (C and D) pRESC8D14 and pRESC8KAI2 were used for the construction of the d14 and kai2 lines. The basic architecture of the vector was the same as shown for the vector described in S1D Fig with d14 and kai2 fragments replacing the concatenated max2 fragments. Relative signal intensity of d14#1 and d14#2 (C) and kai2#1 and kai2#2 (D) plants was quantified with the indicated primers. (E) Vector map of pSOL8CCD7 transformation construct used for constructing ccd7 lines and relative signal intensity of ccd7#1 and ccd7#2 plants with indicated primers. Primers for constructing vectors are described in S2 Table. Values for graphs (C-E) are listed in S1 Data. At, A. thaliana; CCD, carotenoid cleavage dioxygenase; d14, dwarf14; max2, more axillary growth 2; Mt, Medicago truncatula; Na, N. attenuata; Os, O. sativa; Ph, P. hybrida; Ps, Pisum sativum; Pt, Populus trichocarpa; Sl, S. lycopersicum. (TIF) [file pbio.3000830.s002.tif]

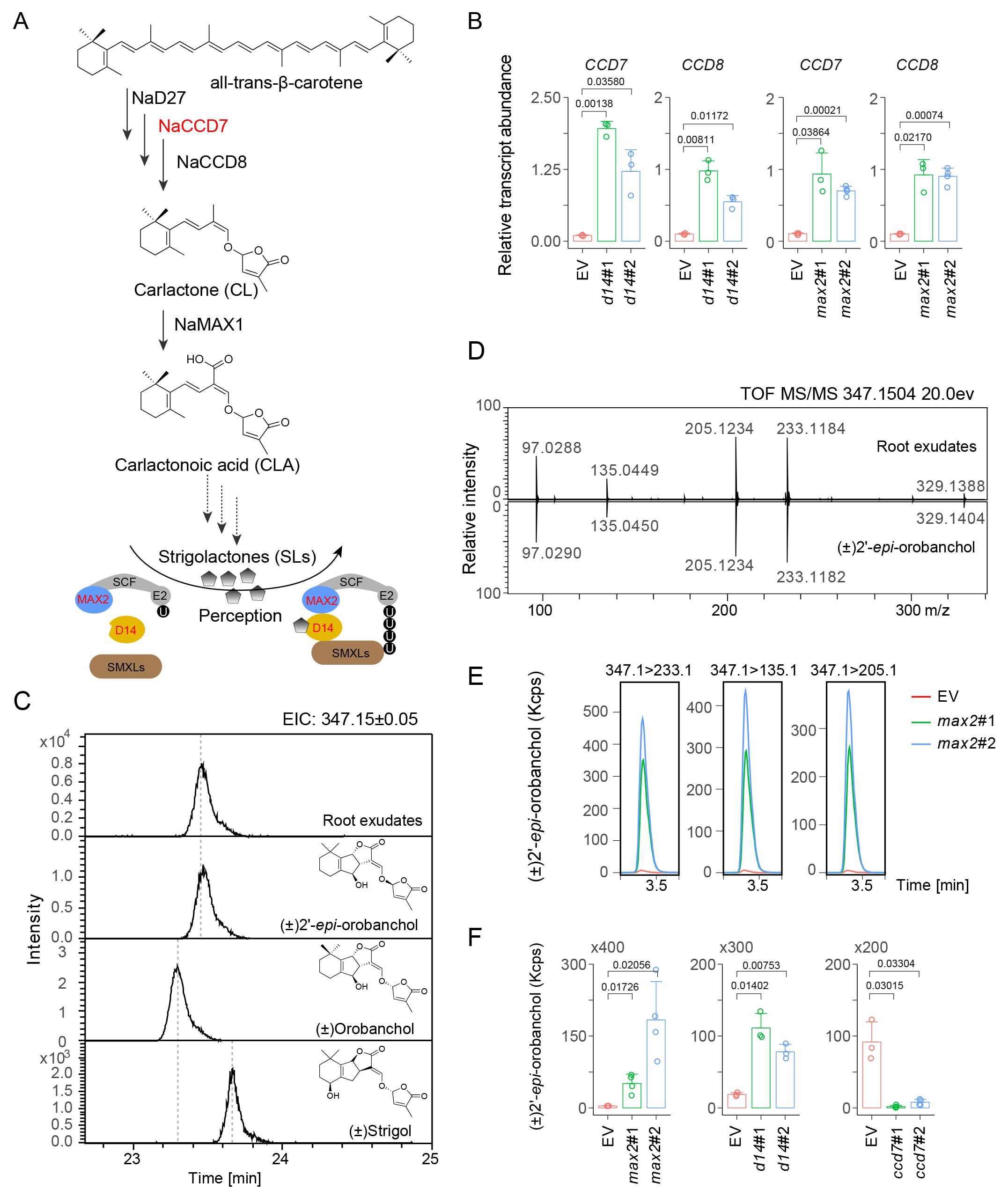

Supplement: S3 Fig — (A) A scheme of SL biosynthesis and perception in N. attenuata. (B) Relative transcript abundance of CCD7 and CCD8 in roots of EV, d14, and max2 plants (±SE, n = 3–4). (C) Representative chromatograms of root exudates, (±)-2′-epi-orobanchol, (±) orobanchol, and (±) strigol analyzed under positive ionization mode. EIC: 347.15 ± 0.05. (D) Comparison of fragmentation patterns of root exudates and (±)-2′-epi-orobanchol by high-resolution tandem MS. (E) Representative chromatograms of (±)-2′-epi-orobanchol in root exudates, as analyzed by targeted UHPLC-triple quadrupole-MS metabolomics; different MRM ions were listed on the top of the panel. (F) (±)-2′-epi-orobanchol levels (Kcps) in root exudates of hydroponic EV, max2, d14, and ccd7 plants (±SE, n = 3–4) were analyzed by targeted UHPLC-triple quadrupole-MS metabolomics. Quantifier ion (m/z) 347.1 > 233.1 was selected to achieve maximum signal intensity. Three independent experiments were performed at different developmental stages (two-tailed Student t test). Values for graphs (B) and (F) are listed in S1 Data. ccd, carotenoid cleavage dioxygenase; d14, dwarf 14; EIC, extracted ion chromatogram; EV, empty vector; Kcps, kilo counts per second; max2, more axillary growth 2; MRM, multiple reaction monitoring; SL, strigolactone; UHPLC, ultra-high-performance liquid chromatography. (TIF) [file pbio.3000830.s003.tif]

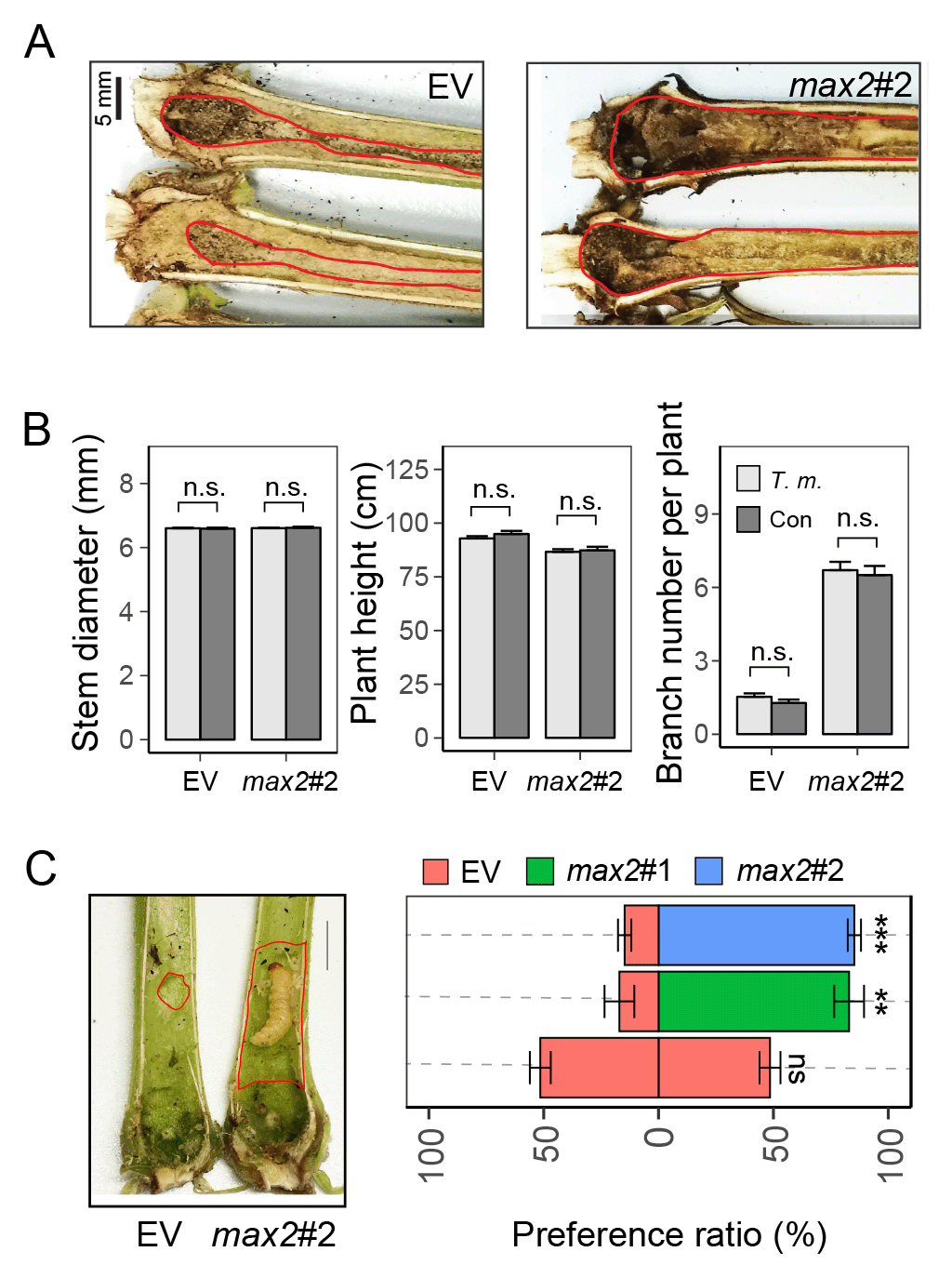

Supplement: S4 Fig — (A) Representative phenotypes of EV and max2#2 pith after attack by T. mucorea larvae for 3 weeks. The attacked area in the pith is outlined with red lines. Scale bar, 5 mm. (B) Stem diameter, plant height, and branch number per plant of EV and max2#2 plants without or with T. mucorea larvae attack (±SE, n = 10–20). (C) Representative images and results of T. mucorea attacked pith from pith-preference bioassays. Scale bar, 5 mm. For the preference bioassays, larvae were placed between two stem halves of either EV and EV, EV and max2#1, or EV and max2#2 plants. The amount of pith damaged in both halves was quantified after 36 hours (±SE, 3 replicates, each replicate includes 8–10 plants) (**P < 0.01; ***P < 0.001; two-tailed Student t test). Values for graphs (B) and (C) are listed in S1 Data. EV, empty vector; max2, more axillary growth 2; n.s., not significant. (TIF) [file pbio.3000830.s004.tif]

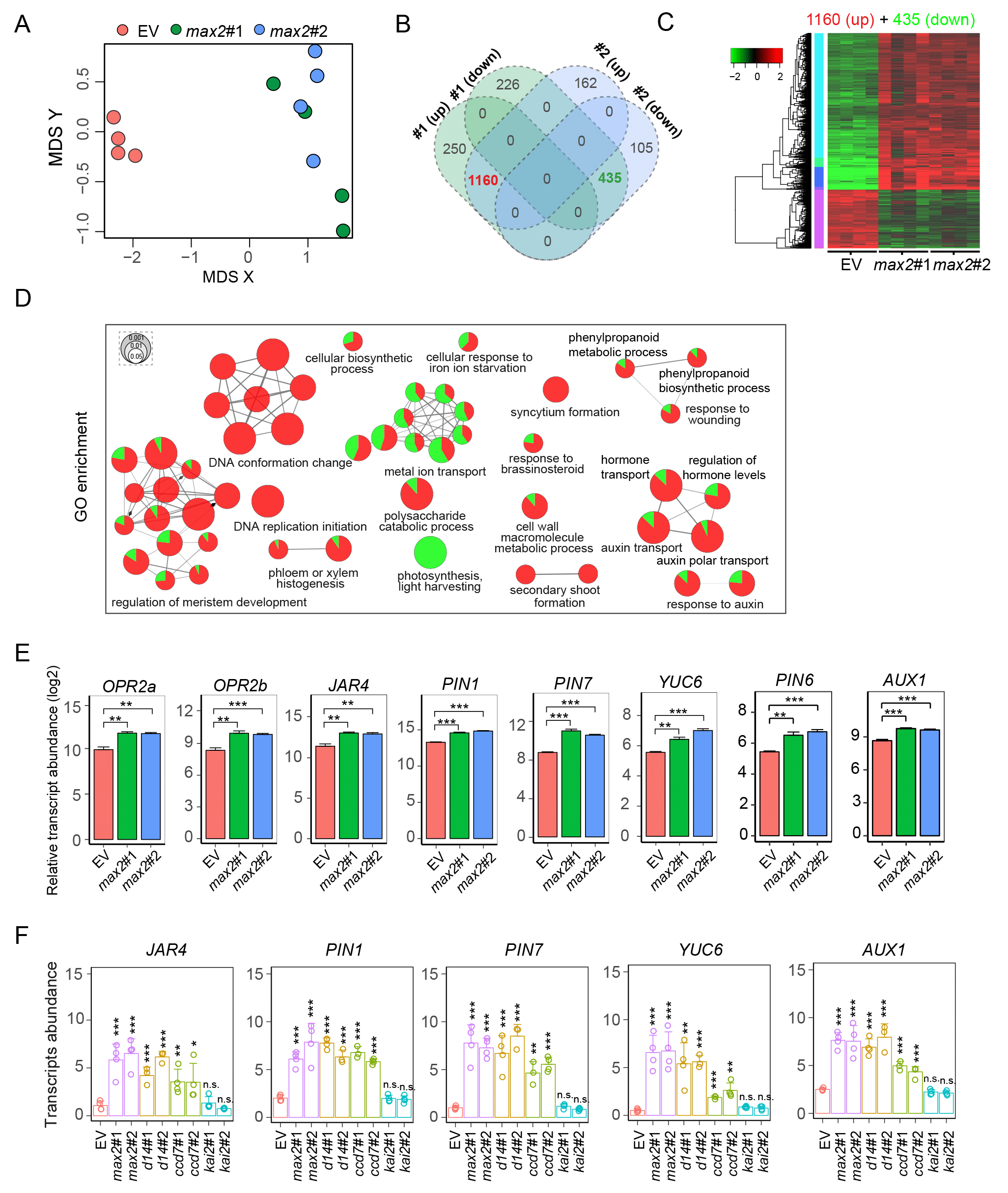

Supplement: S5 Fig — (A) MDS plot of the gene expression data in the RSJs of EV (red), max2#1 (green), and max2#2 (blue) plants. Microarray analysis was performed for gene expression. Each line included four replicate plants. (B) Venn diagrams showing overlap among up-regulated and down-regulated genes in the RSJs of max2#1 and max2#2 plants. (C) Heatmap presents the expression of up-regulated and down-regulated genes in the RSJs of EV, max2#1, and max2#2 plants. The color gradient represents the relative sequence abundance. (D) GO enrichment of 1,160 up-regulated and 435 down-regulated genes in the RSJs of both max2#1 and max2#2 plants. (E) Relative transcript abundance of JA-related genes OPR2a, OPR2b, JAR4, and auxin-related genes PIN1, PIN7, YUC6, PIN6, and AUX1. Expression levels were analyzed from microarray data (±SE, n = 4). (F) Relative transcript abundance of JAR4, PIN1, PIN7, YUC6, and AUX1 in RSJ of indicated plants. Expression levels were analyzed by RT-qPCR (±SE, n = 4) (*P < 0.05; **P < 0.01; ***P < 0.001; two-tailed Student t test). Values for graphs (E) and (F) are listed in S1 Data. AUX1, AUXIN RESISTANT 1; EV, empty vector; GO, Gene Ontology; JA, jasmonate; JAR4, JASMONIC ACID RESISTANT 4; MAX2, more axillary growth 2; MDS, multidimensional scaling; OPR, OPDA reductase; PIN, PIN-FORMED; RSJ, root–shoot junction; YUC6, YUCCA 6. (TIF) [file pbio.3000830.s005.tif]

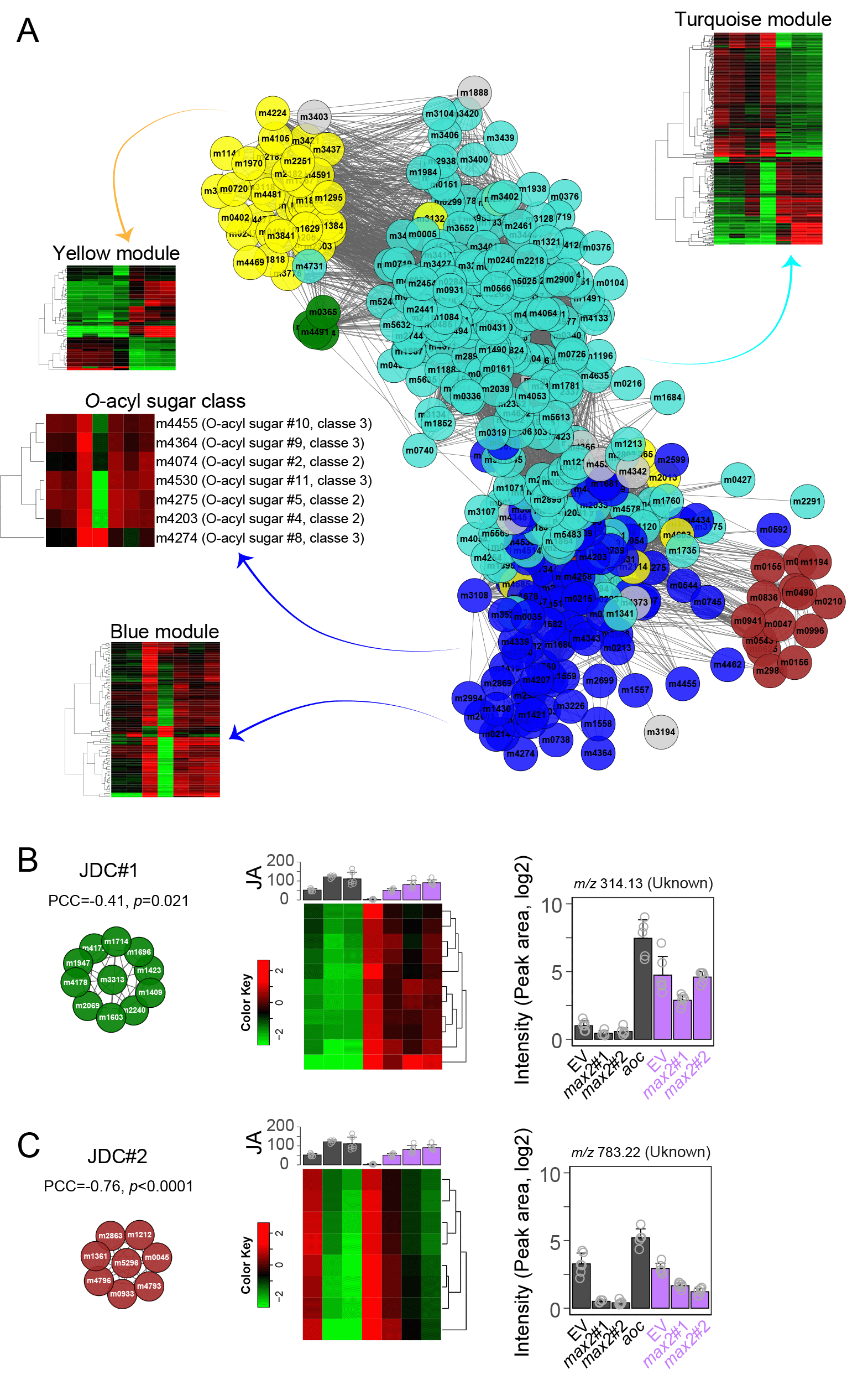

Supplement: S6 Fig — (A) The reciprocal interactions amongst features (TOM > 0.3) was exported as visualization input by Cytoscape. Based on exported features in network, the coexpression patterns of the different modules are illustrated by heatmaps; the class of annotated O-acyl sugar specialized metabolites was specifically extracted from the blue module. Node colors indicate computed modules by WGCNA. (B and C) Extracted subnetworks of JDC#1 and JDC#2 visualized by Cytoscape (left panels) and correlations with JA contents with each subnetwork were calculated as Pearson’s correlation coefficients and their P values. Bar charts display the representative compounds from each subnetwork across all samples from two batches (dark gray: first batch; purple: second batch). Values for graphs (B) and (C) are listed in S1 Table. JA, jasmonate; JDC, JA-dependent cluster; TOM, topological overlap matrix; WGCNA, weighted correlation network analysis. (TIF) [file pbio.3000830.s006.tif]

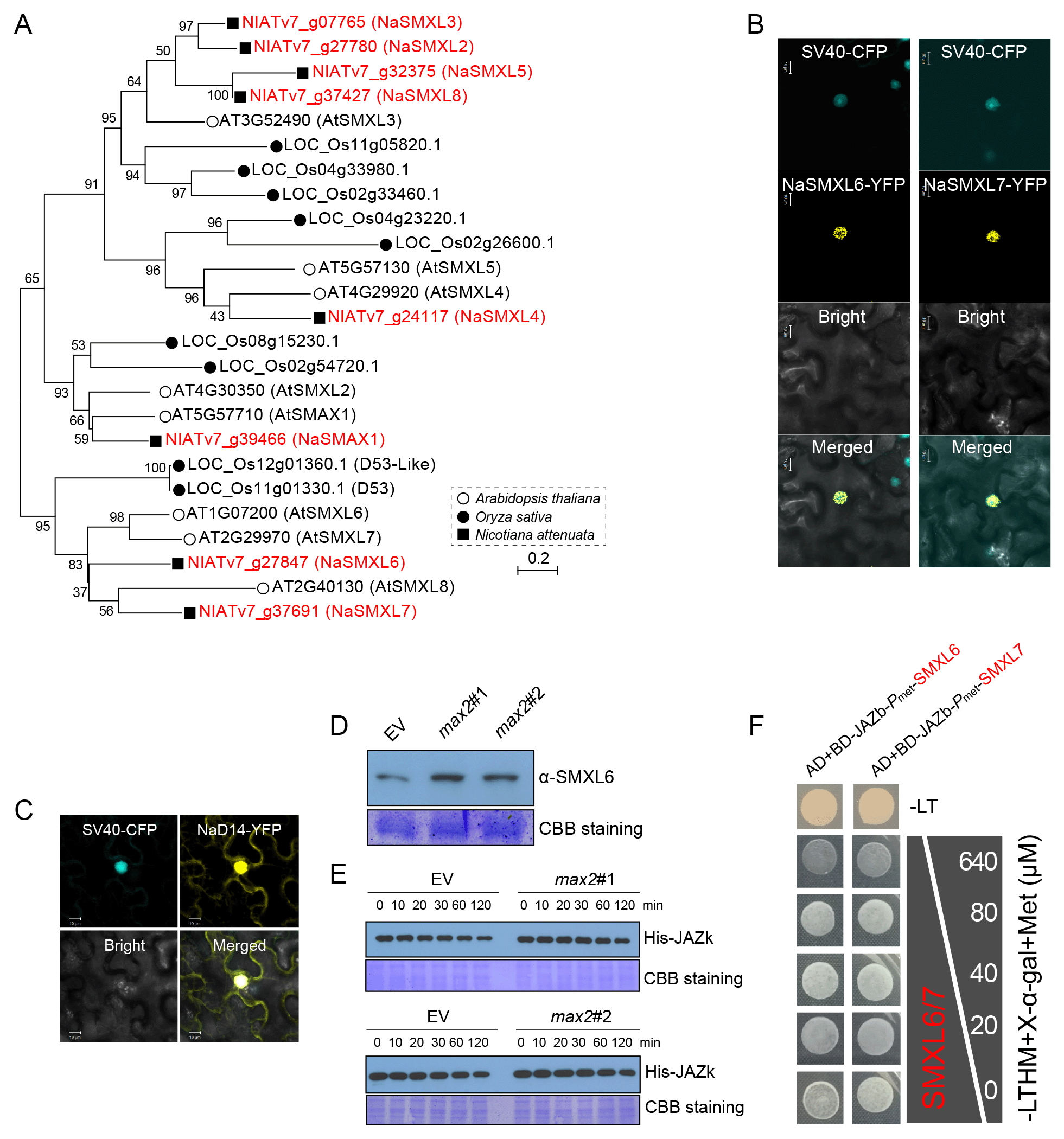

Supplement: S7 Fig — (A) Extended phylogenetic analysis for Fig 7B of SMXL gene family from Na (red), At, and Os. (B) Subcellular localization of NaSMXL6 and NaSMXL7. NaSMXL6-YFP or NaSMXL7-YFP were coinoculated with SV40-CFP and transiently expressed in N. benthamiana leaves for 72 hours. Scale bar, 10 μm. (C) Subcellular localization of NaD14. NaD14-YFP and SV40-CFP were coinoculated and transiently expressed in N. benthamiana leaves for 72 hours. Scale bar, 10 μm. (D) Protein levels of NaSMXL6 in leaves of EV, max2#1, and max2#2 plants. (E) JAZk degradation in EV and max2 (#1, #2) crude proteins. Purified His-JAZk was incubated in EV or max2 crude proteins extracted from the pith for the indicated times. His-JAZk were detected by anti-His. The CBB staining presents protein loading levels. (F) Extended results for Fig 7K of the interference of SMXL6/7 in the interactions of JAZb and MYC2a/b as revealed by Y3H assays. The expression of SMXL6/7 was gradually induced by decreasing concentrations of Met. Raw images for blots are listed in S1 Raw Images. At, A. thaliana; CBB, Coomassie Brilliant Blue; D14, DWARF14; EV, empty vector; JA, jasmonate; JAZ, jasmonate zim-domain; MAX2, more axillary growth 2; Met, methionine; Na, N. attenuata; Os, O. sativa; SMXL, suppressor of max2-like; YFP, yellow fluorescent protein. (TIF) [file pbio.3000830.s007.tif]

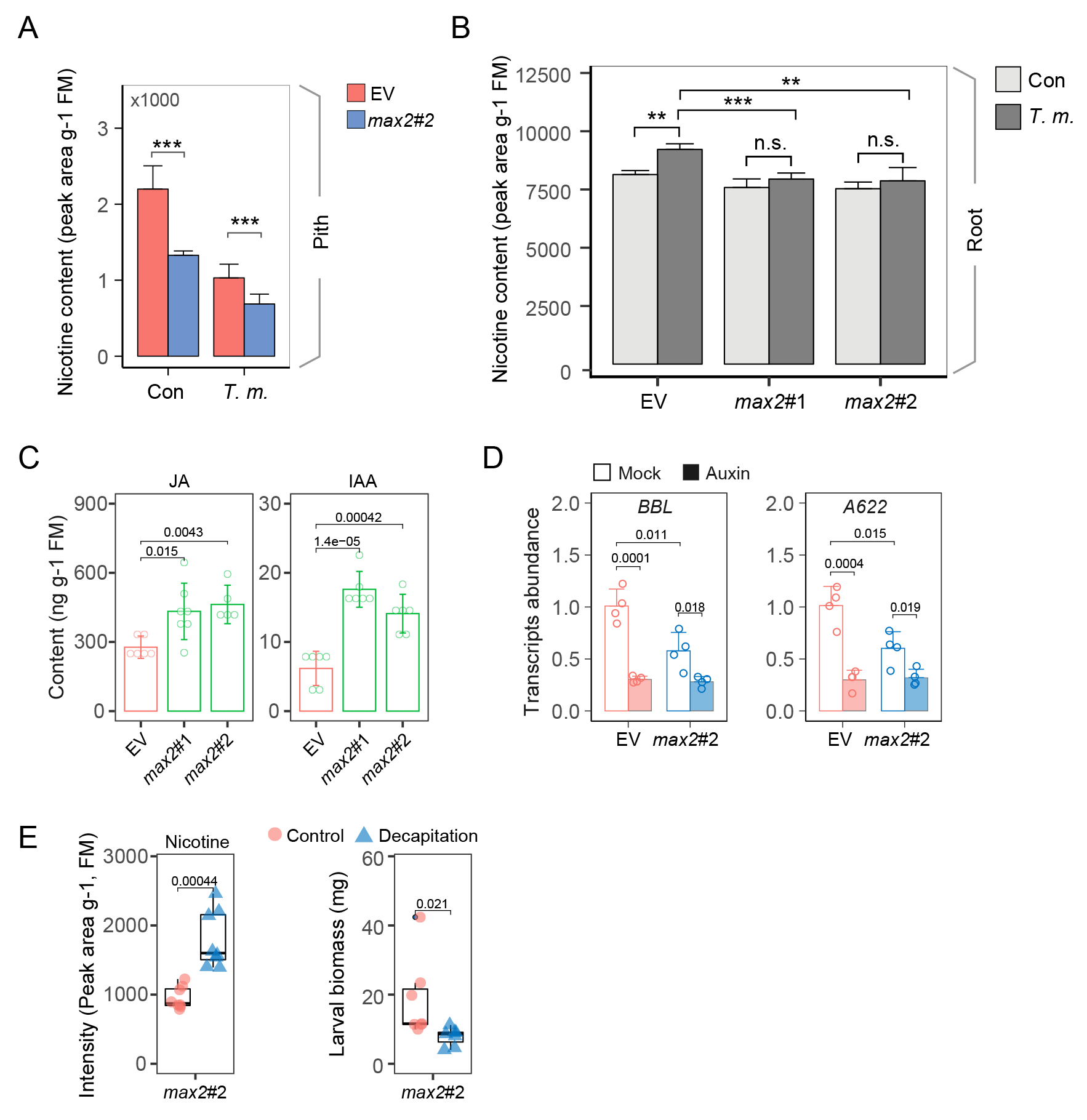

Supplement: S8 Fig — (A) Relative content of nicotine in the pith of EV and max2#2 plants with or without T. mucorea attack for 2 weeks (±SE, n = 5–10). (B) Relative contents of nicotine in roots of EV, max2#1, and max2#2 plants with or without T. mucorea attack (±SE, n = 6–8). (C) Relative contents of JA and IAA in roots of EV, max2#1, and max2#2 plants (±SE, n = 6). (D) Relative transcript abundance of nicotine biosynthetic genes BBL and A622 in EV and max2#2 seedlings with or without 10 μM IAA treatment for 8 hours. Transcripts levels were quantified by RT-qPCR (±SE, n = 4). (E) Nicotine contents in the RSJs of max2#2 plants with or without decapitation treatments for 3 days (n = 8) (two-tailed Student t test). Values for graphs (A–E) are listed in S1 Data. EV, empty vector; IAA, indole-3-acetic acid; JA, jasmonate; MAX2, more axillary growth 2; RSJ, root–shoot junction. (TIF) [file pbio.3000830.s008.tif]
