# Supplementary material for: Strigolactone signaling regulates specialized metabolism in tobacco stems and interactions with stem-feeding herbivores
Source: PLoS Biol. 2020 Aug 18;18(8):e3000830. doi: 10.1371/journal.pbio.3000830 (PMC7478753; doi:10.1371/journal.pbio.3000830)
Supplement: S1 Raw Images — (PDF) [file pbio.3000830.s012.pdf]

Fig 7D

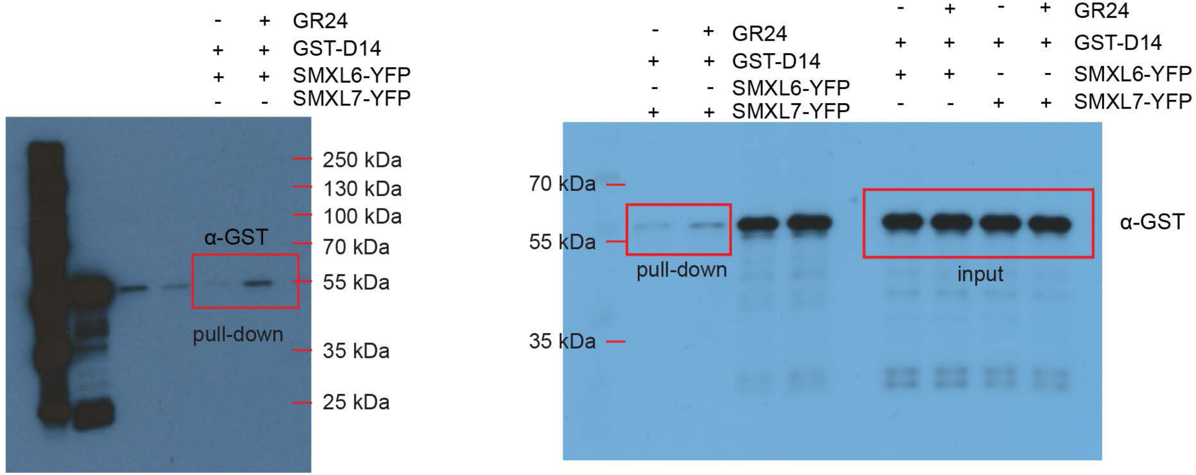

- + GR24
+ + GST-D14
+ + SMXL6-YFP
- - SMXL7-YFP
250 kDa
130 kDa
100 kDa
70 kDa
55 kDa
35 kDa
25 kDa
α-GST
pull-down
- + GR24
+ + GST-D14
- - SMXL6-YFP
+ + SMXL7-YFP
- + - + GR24
+ + + + GST-D14
+ + - - SMXL6-YFP
- - + + SMXL7-YFP
70 kDa
55 kDa
35 kDa
pull-down
input
α-GST

Fig 7G

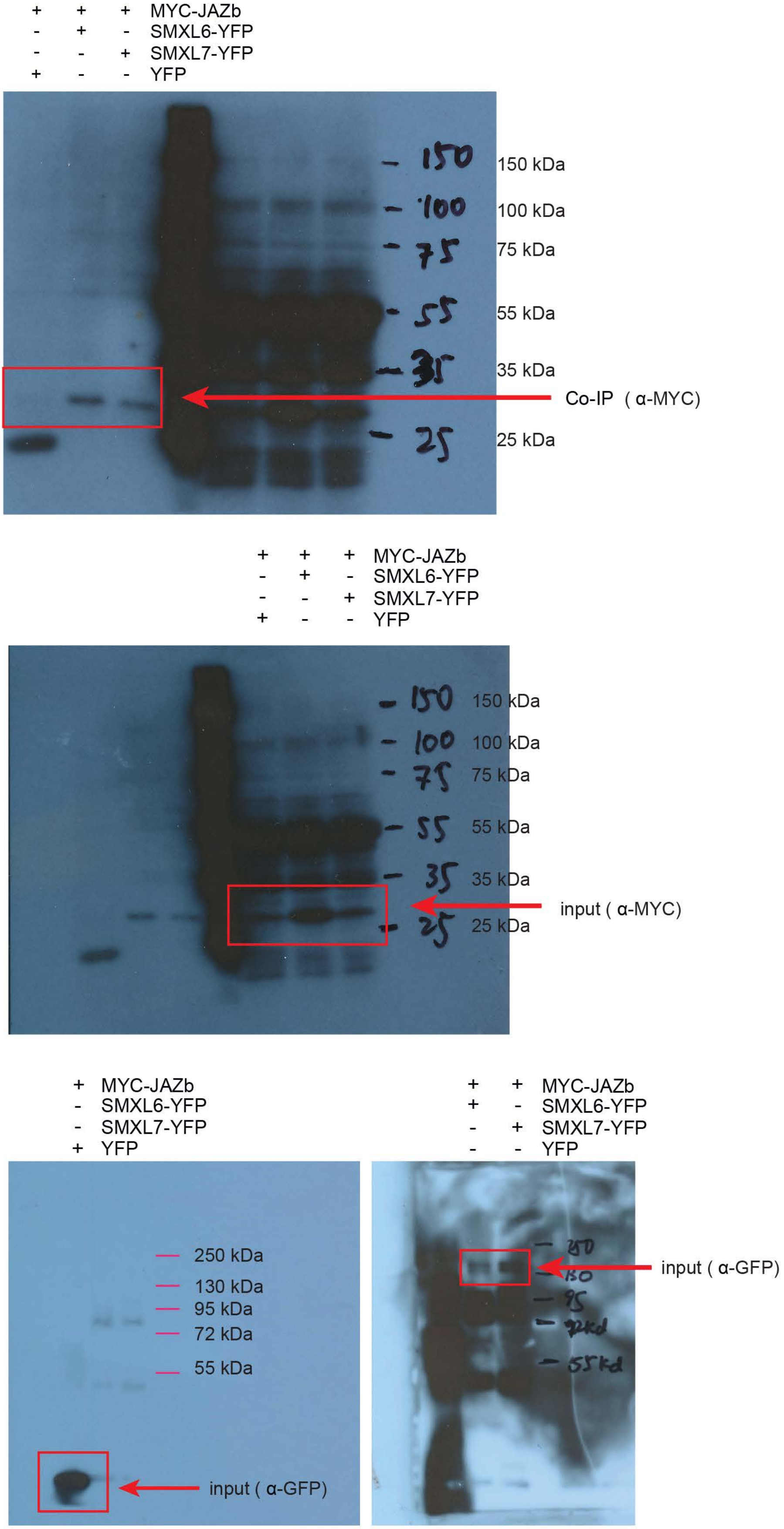

+ + + MYC-JAZb
- + - SMXL6-YFP
- - + SMXL7-YFP
+ - - YFP
150 kDa
100 kDa
75 kDa
55 kDa
35 kDa
Co-IP (α-MYC)
25 kDa
+ + + MYC-JAZb
- + - SMXL6-YFP
- - + SMXL7-YFP
+ - - YFP
150 kDa
100 kDa
75 kDa
55 kDa
35 kDa
input (α-MYC)
25 kDa
+ MYC-JAZb
- SMXL6-YFP
- SMXL7-YFP
+ YFP
250 kDa
130 kDa
95 kDa
72 kDa
55 kDa
input (α-GFP)
+ + MYC-JAZb
+ - SMXL6-YFP
- + SMXL7-YFP
- - YFP
input (α-GFP)

Fig 7H

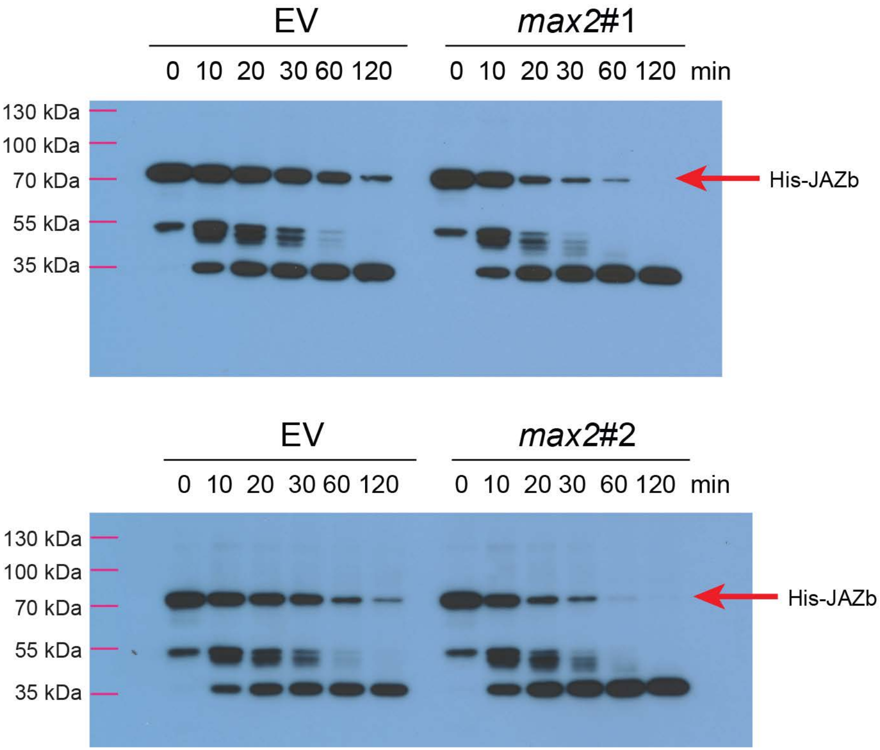
EV
max2#1
0 10 20 30 60 120
0 10 20 30 60 120 min
130 kDa
100 kDa
70 kDa
55 kDa
35 kDa
His-JAZb
EV
max2#2
0 10 20 30 60 120
0 10 20 30 60 120 min
130 kDa
100 kDa
70 kDa
55 kDa
35 kDa
His-JAZb

Fig 7I

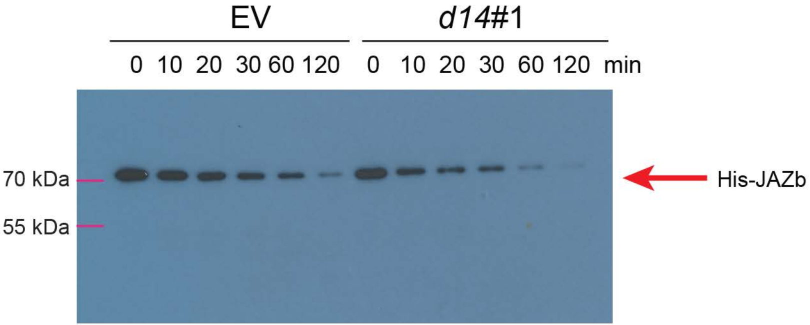
EV
d14#1
0 10 20 30 60 120 0 10 20 30 60 120 min
70 kDa
55 kDa
His-JAZb

Fig 7J

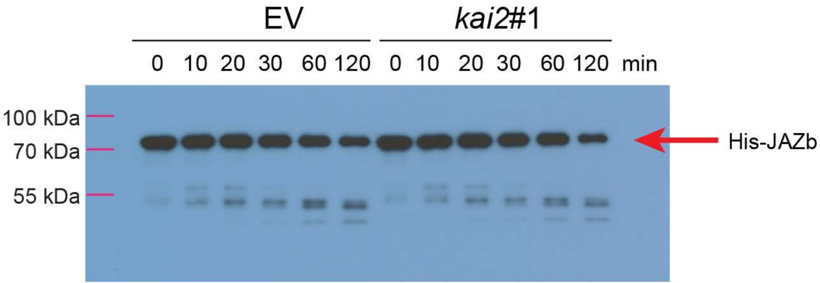
EV
kai2#1
0
10
20
30
60
120
0
10
20
30
60
120
min
100 kDa
70 kDa
55 kDa
His-JAZb

S7D Fig

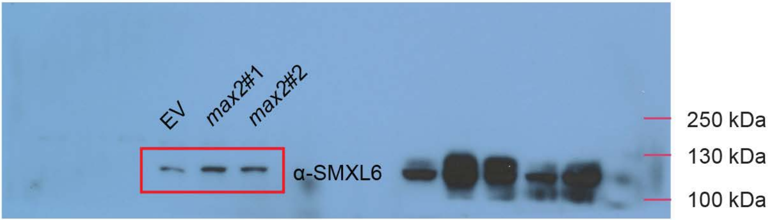

S7E Fig

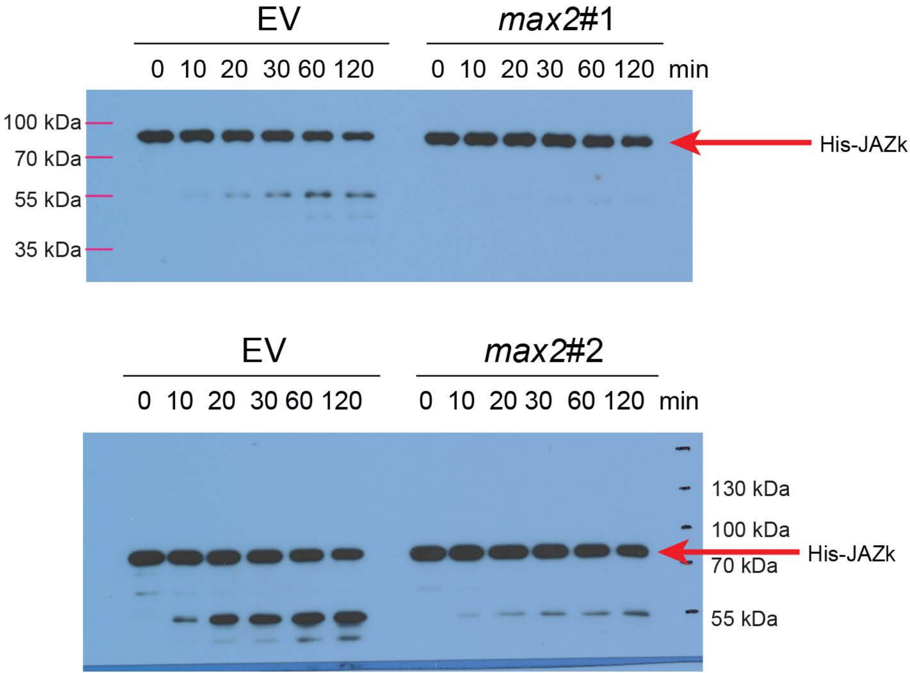
EV
max2#1
0 10 20 30 60 120
0 10 20 30 60 120 min
100 kDa
70 kDa
55 kDa
35 kDa
His-JAZk
EV
max2#2
0 10 20 30 60 120
0 10 20 30 60 120 min
130 kDa
100 kDa
70 kDa
55 kDa
His-JAZk
